# Supplementary material for: High body temperature increases gut microbiota-dependent host resistance to influenza A virus and SARS-CoV-2 infection
Source: Nat Commun. 2023 Jun 30;14:3863. doi: 10.1038/s41467-023-39569-0 (PMC10313692; doi:10.1038/s41467-023-39569-0)
Supplement: Supplementary file 1 — Supplementary Information [file 41467_2023_39569_MOESM1_ESM.pdf]

Supplementary Information for

High body temperature increases gut microbiota-dependent host resistance to influenza A virus and SARS-CoV-2 infection

Nagai, Moriyama, and Ishii et al.

This file includes:

Supplementary Figures 1-37

Supplementary Table 1

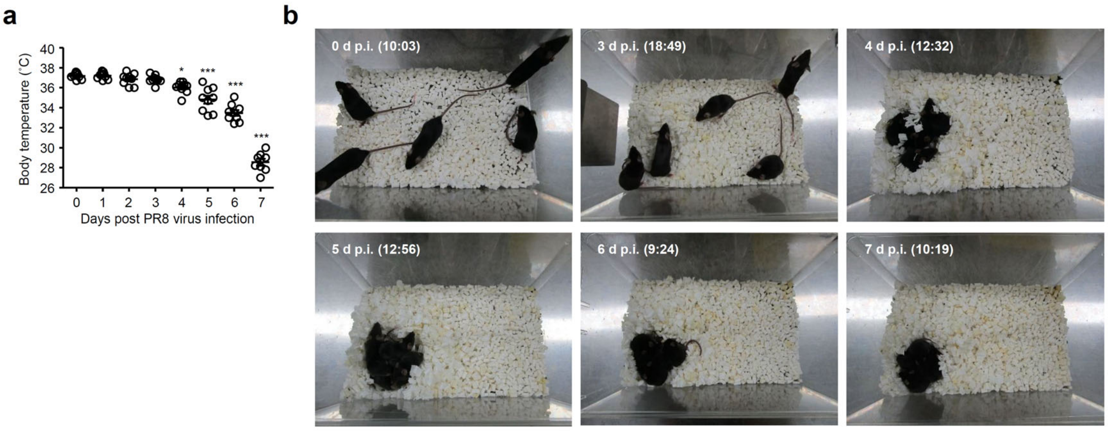

Supplementary Figure 1

Changes of body temperature following influenza virus infection.

a, b, Mice kept at 22 °C were infected intranasally with 1,000 pfu of influenza virus. Body temperatures (**a**; *n* = 9 mice) and activity of infected mice (**b**; *n* = 5 mice) were monitored for 7 days. The numbers in parentheses indicate the time of day at which these pictures were taken (**b**). Data are mean ± s.e.m. Data are representative of two independent experiments. Statistical significance was analysed by two-way analysis of variance (ANOVA) test (**a**). *P<0.05, ***P<0.001.

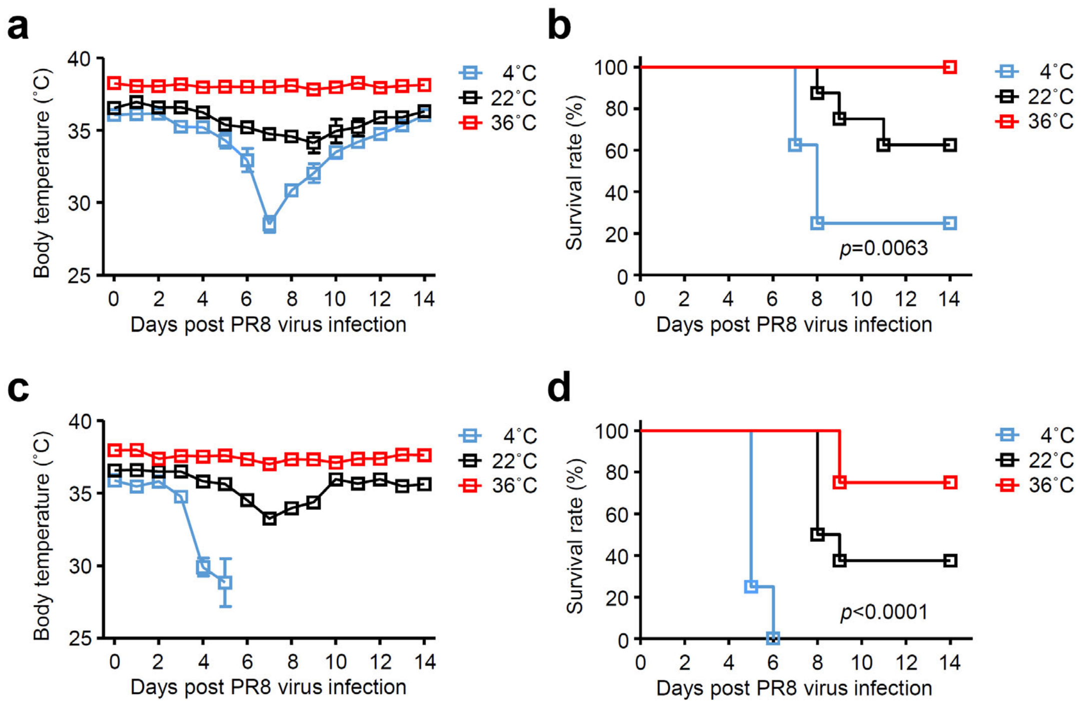

Supplementary Figure 2

Body temperature affects severity of influenza virus infection.

a-d, Mice were kept at 4, 22, or 36 °C for 7 d before influenza virus infection and throughout infection. Mice kept at 4, 22, or 36 °C were infected intranasally with 500 (**a, b**) or 2,000 (**c, d**) pfu of influenza virus. Body temperatures (**a, c**) and mortality (**b, d**) were monitored for 14 days (4 °C, n = 8 mice; 22 °C, n = 8 mice; 36 °C, n = 8 mice). Data are mean ± s.e.m. Data are representative of two independent experiments. Statistical significance was analysed by log-rank (Mantel-Cox) test (**b**, **d**).

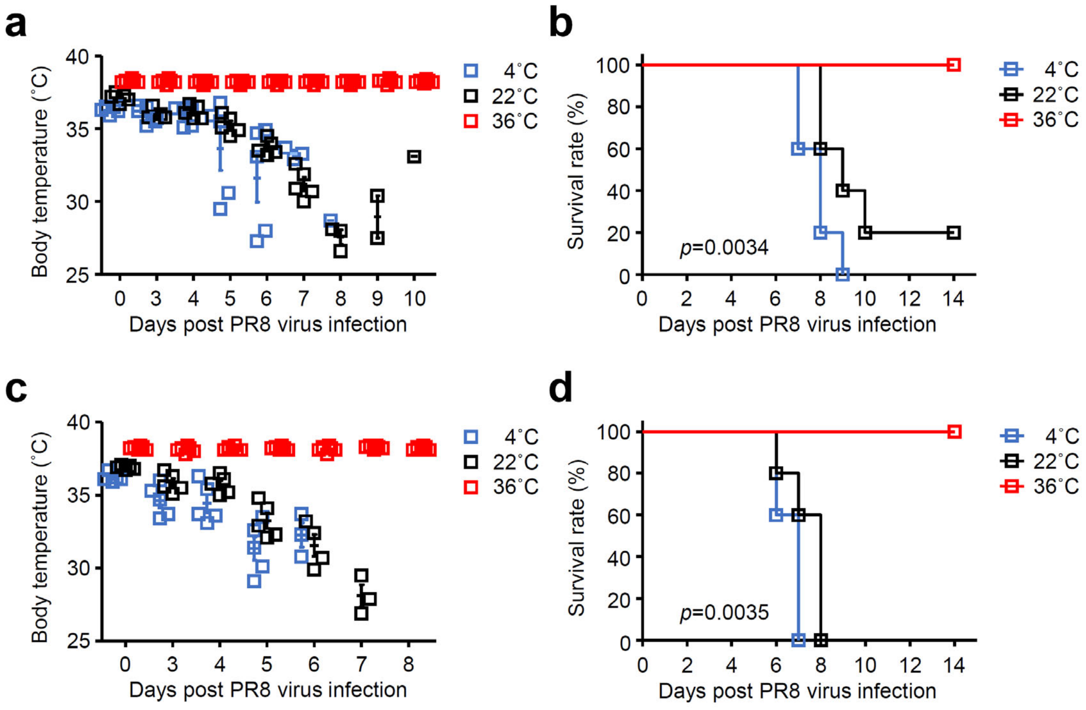

Supplementary Figure 3

Body temperature affects severity of influenza virus infection.

a-d, Mice were kept at 4, 22, or 36 °C for 7 d before influenza virus infection and throughout infection. Mice kept at 4, 22, or 36 °C were infected intratracheally with 1,000 (**a, b**) or 10,000 (**c, d**) pfu of influenza virus. Body temperatures (**a, c**) and mortality (**b, d**) were monitored for 14 days (4 °C, *n* = 5 mice; 22 °C, *n* = 5 mice; 36 °C, *n* = 6 mice). Data are mean ± s.e.m. Data are representative of two independent experiments. Statistical significance was analysed by log-rank (Mantel-Cox) test (**b**, **d**).

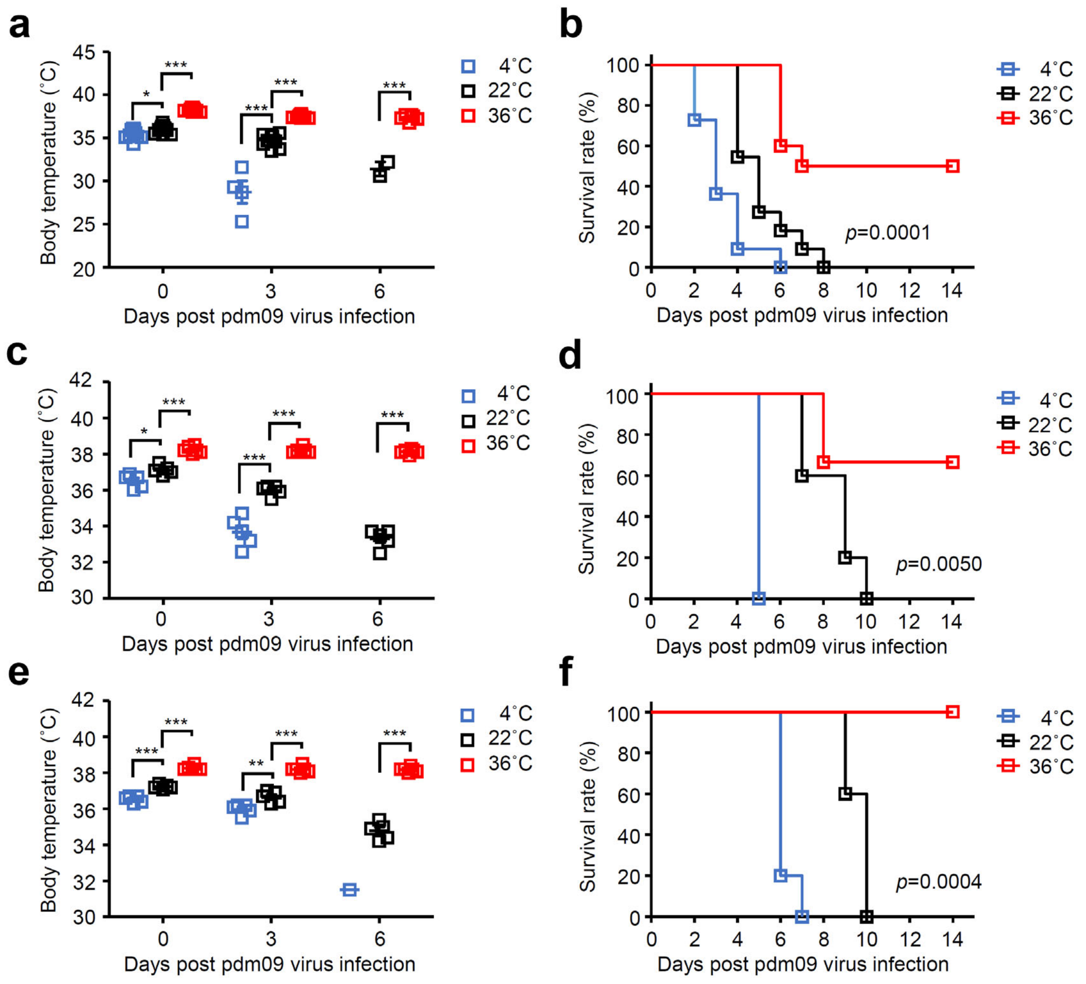

Supplementary Figure 4

Body temperature affects severity of influenza virus pdm09 infection.

a-f, Mice were kept at 4, 22, or 36 °C for 7 d before influenza virus infection and throughout infection. Mice kept at 4, 22, or 36 °C were infected intranasally with 3 $\times 10^4$ pfu (**a, b**; 4 °C, *n* = 11 mice; 22 °C, *n* = 11 mice; 36 °C, *n* = 10 mice), 3,000 pfu (**c, d**; 4 °C, *n* = 5 mice; 22 °C, *n* = 5 mice; 36 °C, *n* = 6 mice), or 500 pfu (**e, f**; 4 °C, *n* = 5 mice; 22 °C, *n* = 5 mice; 36 °C, *n* = 6 mice) of a human isolate of the 2009 pandemic influenza A virus strain A/Narita/1/2009 (pdm09). Body temperatures (**a, c, e**) and mortality (**b, d, f**) were monitored for 14 days. Data are mean ± s.e.m. Data are representative of two independent experiments. Statistical significance was analysed by two-way analysis of variance (ANOVA) test (**a**, **c, e**) or log-rank (Mantel-Cox) test (**b**, **d, f**). *P<0.05, ***P<0.001.

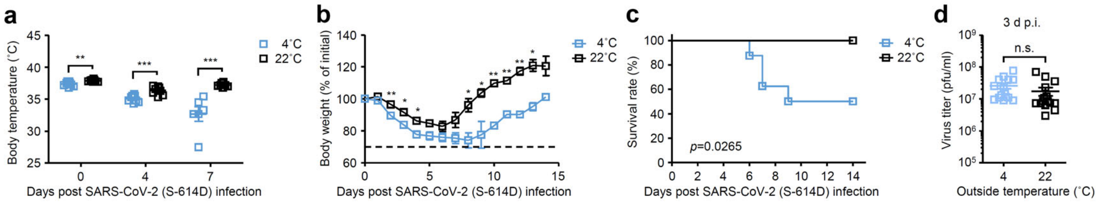

Supplementary Figure 5

Effects of outside temperature on severity of SARS-CoV-2 infection.

a-d, Syrian hamsters were kept at 4 or 22 °C for 7 d before original SARS-CoV-2 (S-614D) infection and throughout infection. Hamsters kept at 4 or 22 °C were infected intranasally with 1.5×10^6 pfu of an original SARS-CoV-2 (S-614D) strain. Body temperatures (**a**; 4 °C, *n* = 8 hamsters; 22 °C, *n* = 8 hamsters), wight loss (**b**; 4 °C, *n* = 3 hamsters; 22 °C, *n* = 3 hamsters), mortality (**c**; 4 °C, *n* = 8 hamsters; 22 °C, *n* = 8 hamsters), and virus titer in the lung wash (**d**; 4 °C, *n* = 15 hamsters; 22 °C, *n* = 15 hamsters) were measured on indicated days after challenge. The dashed line indicates the limit of endpoint. Data are mean ± s.e.m. Data are representative of two independent experiments (**a**, **b**) or are pooled from two (**c**) or three (**d**) independent experiments. Statistical significance was analysed by two-tailed unpaired Student's *t*-test (**a**, **b**, **d**) or log-rank (Mantel-Cox) test (**c**). *P<0.05, **P<0.01, ***P<0.001, n.s., not significant (**a**; 0 d p.i., p=0.002226; 4 d p.i., p=0.00082; 7 d p.i., p=0.000529; **b**; 2 d p.i., p=0.001506; 3 d p.i., p=0.0164; 4 d p.i., p=0.0184; 8 d p.i., p=0.0237; 9 d p.i., p=0.0412; 10 d p.i., p=0.00261; 11 d p.i., p=0.0019; 12 d p.i., p=0.00387; 13 d p.i., p=0.0152).

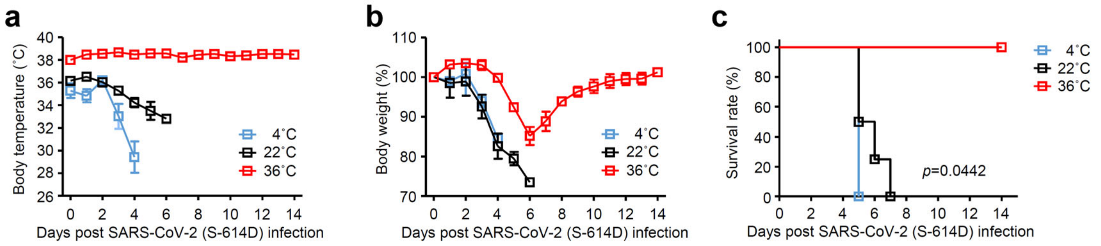

Supplementary Figure 6

Body temperature affects severity of SARS-CoV-2 infection.

a-c, K18-hACE2 mice were kept at 4, 22, or 36 °C for 7 d before original SARS-CoV-2 (S-614D) infection and throughout infection. K18-hACE2 mice kept at 4, 22, or 36 °C were infected intranasally with 5×10^4 pfu of an original SARS-CoV-2 (S-614D) strain. Body temperatures (**a**), weight loss (**b**), and mortality (**c**) were monitored for 14 days (4 °C, *n* = 5 mice; 22 °C, *n* = 4 mice; 36 °C, *n* = 4 mice). Data are mean ± s.e.m. Data are representative of two independent experiments. Statistical significance was analysed by log-rank (Mantel-Cox) test (**c**).

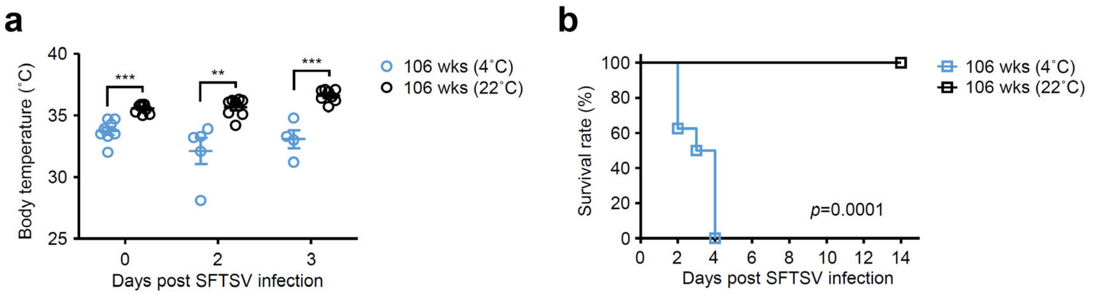

Supplementary Figure 7

Effects of outside temperature on severity of SFTSV infection.

a, b, One hundred six-weeks-old mice were kept at 4 or 22 °C for 7 d before SFTSV infection and throughout infection. 106-weeks-old mice kept at 4 or 22°C were infected intravenously with 5×10^6 $TCID_{50}$ of SFTSV. Body temperatures (**a**) and mortality (**b**) were measured on indicated days after challenge (4 °C, *n* = 8 mice; 22 °C, *n* = 8 mice). Data are mean ± s.e.m. Data are representative of two independent experiments. Statistical significance was analysed by two-tailed unpaired Student's *t*-test (**a**) or log-rank (Mantel-Cox) test (**b**). * ***P*<0.01, ****P*<0.001 (**a**; 0 d p.i., *p*=0.00011; 2 d p.i., *p*=0.001876; 3 d p.i., *p*=0.0000852).

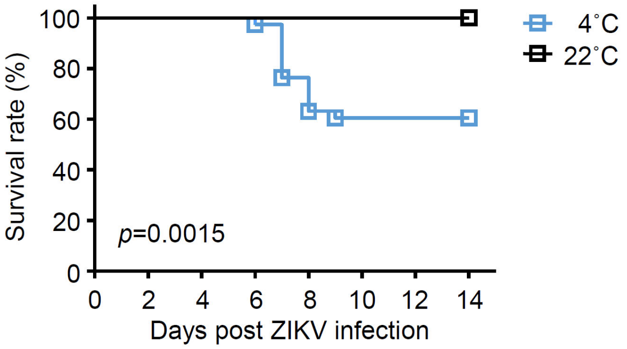

Supplementary Figure 8

Effects of outside temperature on severity of ZIKV infection.

Mice were kept at 4 or 22 °C for 7 d before ZIKV infection and throughout infection. Mice kept at 4 or 22°C were infected intravenously with 1.5×10^7 pfu of ZIKV. Mortality was monitored for 14 days (4 °C, *n* = 38 mice; 22 °C, *n* = 20 mice). Data are pooled from three independent experiments. Statistical significance was analysed by log-rank (Mantel-Cox) test.

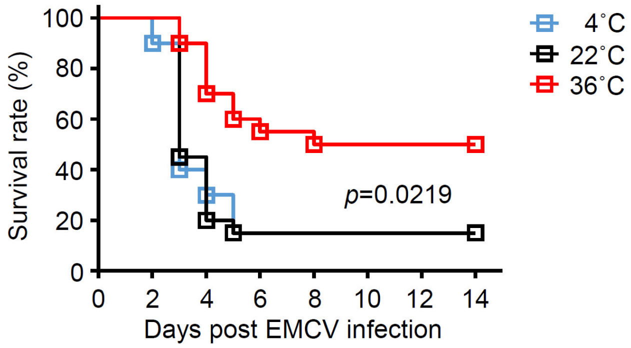

Supplementary Figure 9

Effects of outside temperature on severity of EMCV infection.

Mice were kept at 4, 22, or 36 °C for 7 d before EMCV infection and throughout infection. Mice kept at 4, 22, or 36 °C were infected intraperitoneally with 100 pfu of EMCV. Mortality was monitored for 14 days (4 °C, *n* = 20 mice; 22 °C, *n* = 20 mice; 36 °C, *n* = 20 mice). Data are pooled from two independent experiments. Statistical significance was analysed by log-rank (Mantel-Cox) test.

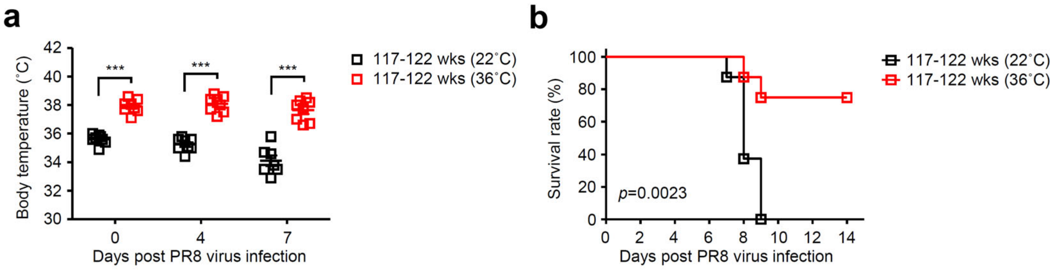

Supplementary Figure 10

High-heat exposure of aged mice had improved survival after influenza virus infection.

a, b, One hundred seventeen- to 122-weeks-old mice were kept at 22 or 36 °C for 7 d before influenza virus infection and throughout infection. Mice kept at 22 or 36°C were infected intranasally with 1,000 pfu of influenza virus. Body temperatures (**a**) and mortality (**b**) were measured on indicated days after challenge (22 °C, *n* = 8 mice; 36 °C, *n* = 8 mice). Data are mean ± s.e.m. Data are representative of two independent experiments. Statistical significance was analysed by two-tailed unpaired Student's *t*-test (**a**) or log-rank (Mantel-Cox) test (**b**). ****P*<0.001 (**a**; 0 d p.i., *p*=0.0000000248; 4 d p.i., *p*=0.0000000304; 7 d p.i., *p*=0.000002735).

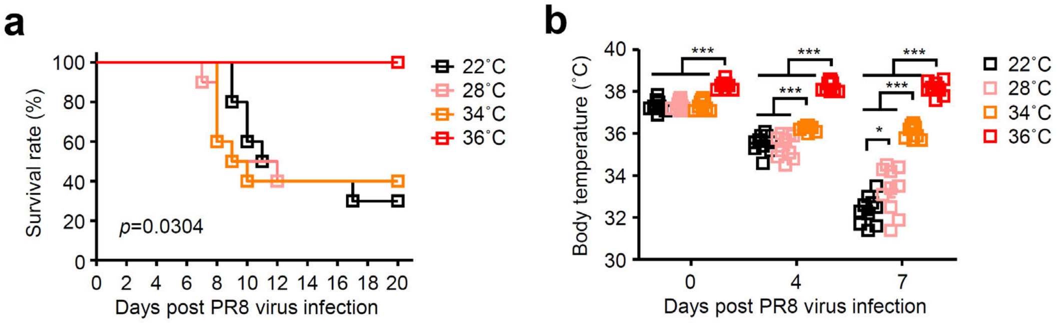

Supplementary Figure 11

Effects of outside temperature on severity of influenza virus infection.

a, b, Mice were kept at 22, 28, 34, or 36 °C for 7 d before influenza virus infection and throughout infection. Mice kept at 22, 28, 34, or 36 °C were infected intranasally with 500 pfu of influenza virus. Mortality (**a**) and body temperatures (**b**) were measured on indicated days after challenge (22 °C, *n* = 10 mice; 28 °C, *n* = 10 mice; 34 °C, *n* = 10 mice; 36 °C, *n* = 10 mice). Data are pooled from two independent experiments. Statistical significance was analysed by log-rank (Mantel-Cox) test (**a**) or two-way analysis of variance (ANOVA) test (**b**). **P*<0.05, ****P*<0.001.

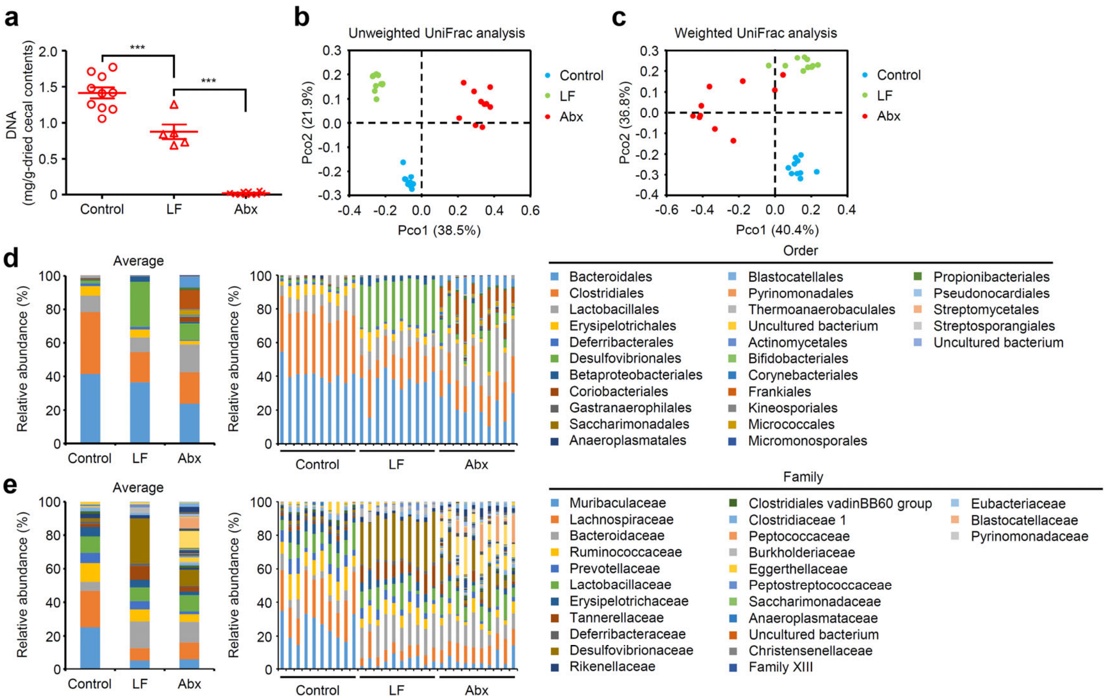

Supplementary Figure 12

Analysis of gut microbiota composition in high heat-exposed control, LF-fed, or Abx-treated mice.

Control, LF-fed, or Abx-treated mice were kept at 36 °C for 7 d. **a,** The amounts of DNA extracted from cecal contents were measured by NanoDrop (control, *n* = 10 mice; LF-fed, *n* = 5 mice; Abx-treated, *n* = 10 mice). **b, c,** Principal coordinates analysis (PCoA) based on Unweighted (b) or Weighted UniFrac analysis (c) on amplicon sequence variant (ASV). Each symbol represents a single sample of cecal content of high heat-exposed control (*n* = 10), LF-fed (*n* = 10), or Abx-treated (*n* = 10) mice. **d, e,** Comparison of order- (**d**) or family- (**e**) level proportional abundance of cecal contents of high heat-exposed control (*n* = 10), LF-fed (*n* = 10), or Abx-treated (*n* = 10) mice. Data are mean ± s.e.m. (**a**) Statistical significance was analysed by two-way analysis of variance (ANOVA) test (**a**). ***P<0.001.

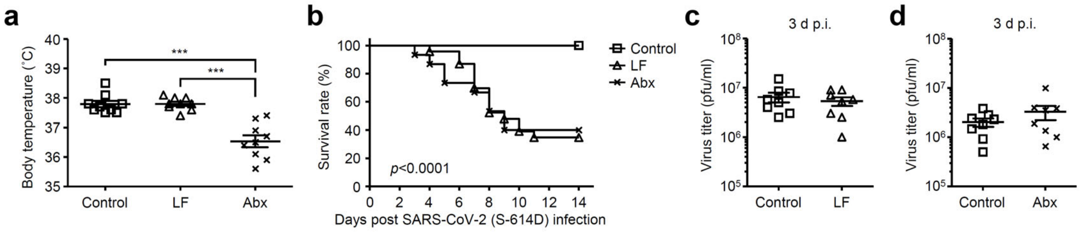

Supplementary Figure 13

Gut microbiota-derived metabolites protect hamsters from SARS-CoV-2 infection.

a-d, Six-weeks-old hamsters were fed a low fiber (LF)-diet (AIN93G) or antibiotics (Abx) in drinking water for 2 weeks before SARS-CoV-2 infection. Body temperature of LF-fed, Abx-treated, and control uninfected hamsters kept at 22 °C were measured (**a**; control, *n* = 9 hamsters; LF, *n* = 9 hamsters; Abx, *n* = 9 hamsters). LF-fed, Abx-treated, and control hamsters kept at 22 °C were infected intranasally with 1.5×10^6 pfu of an original SARS-CoV-2 (S-614D) strain. Mortality (**b**; control, *n* = 27 hamsters; LF, *n* = 23 hamsters; Abx, *n* = 15 hamsters) and virus titer in the lung wash (**c, d**; control, *n* = 8 hamsters; LF, *n* = 8 hamsters; Abx, *n* = 8 hamsters) were measured on indicated days after challenge. Data are mean ± s.e.m. Data are representative of two independent experiments (**a**, **c**, **d**) or are pooled from three (**b**) independent experiments. Statistical significance was analysed by two-way analysis of variance (ANOVA) test (**a**), log-rank (Mantel-Cox) test (**b**), or two-tailed unpaired Student's *t*-test (**c**, **d**). ***$P<0.001$.

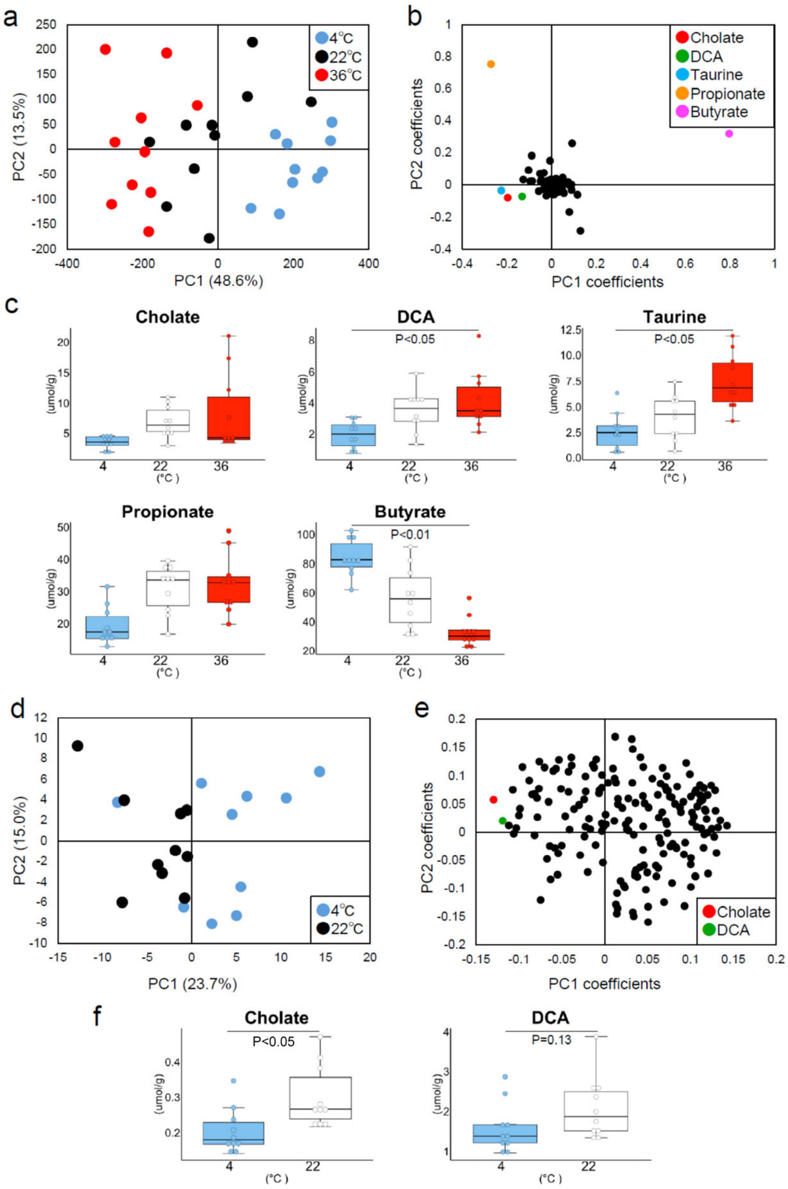

Supplementary Figure 14

Effects of outside temperature on the cecal metabolome profiles of mice and hamsters.

a, b, PCA plot of the cecal metabolome profiles in mice normalized by Pareto (**a**) and loading scatter plot (**b**). **c,** Box plots indicating cecal amounts of metabolites in mice that had |PC1 coefficient values| > 0.13 in PCA. Significant differences are indicated based on Tukey–Kramer test. **d, e,** PCA plot of the cecal metabolome profiles in Syrian hamster normalized by unit variance (**d**) and loading scatter plot (**e**). **f,** Box plots indicating cecal amounts of metabolites in Syrian hamster that had PC1 coefficient values < -0.115 in PCA. Statistical significance was analysed by two-tailed unpaired Student's *t*-test (**c**; DCA,

p=0.002845; Taurine, *p*=0.000146; Butyrate, *p*=0.000000009885; **f**; Cholate, *p*=0.0121; DCA, *p*=0.130155). The centre line denotes the median value (50th percentile), while the white box contains the 25th to 75th percentiles of dataset. The black whiskers mark the 5th and 95th percentiles (**c, f**).

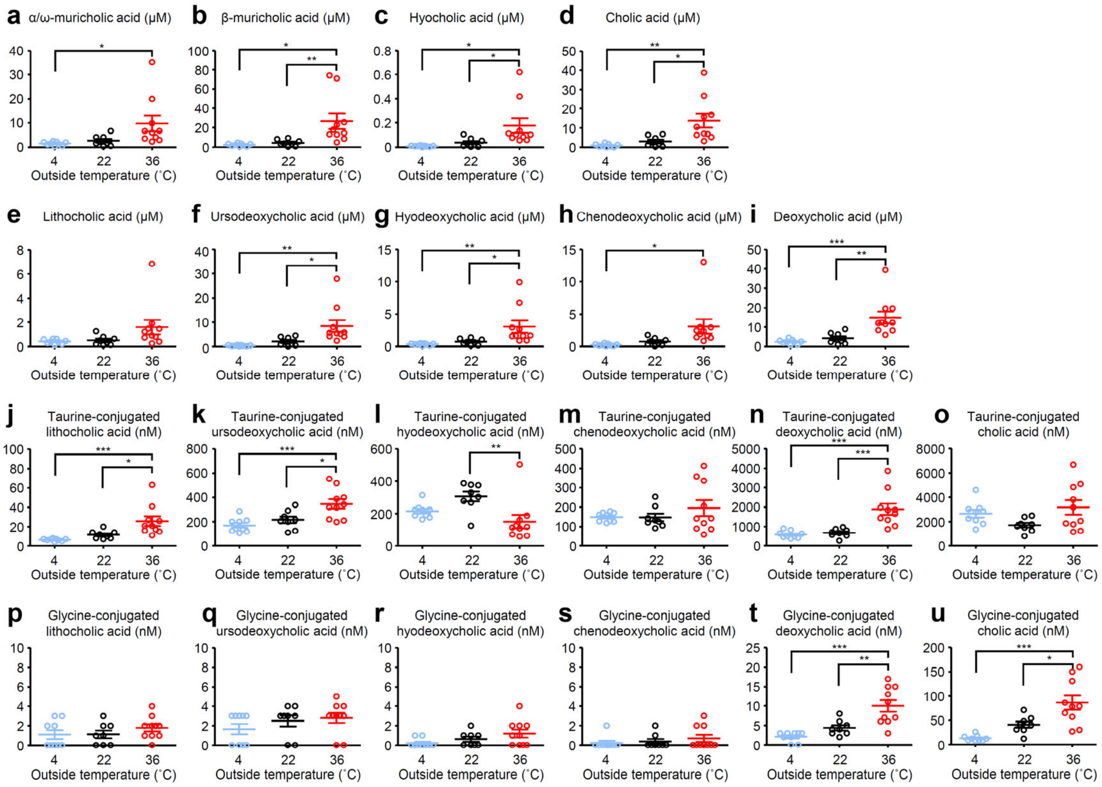

Supplementary Figure 15

The levels of bile acids in serum.

a-u, Mice were kept at 4, 22, or 36 °C for 7 d. The levels of bile acids in serum were measured (4 °C, *n* = 9 mice; 22 °C, *n* = 8 mice; 36 °C, *n* = 10 mice). Data are mean ± s.e.m. Statistical significance was analysed by two-way analysis of variance (ANOVA) test. **P*<0.05, ***P*<0.01, ****P*<0.001.

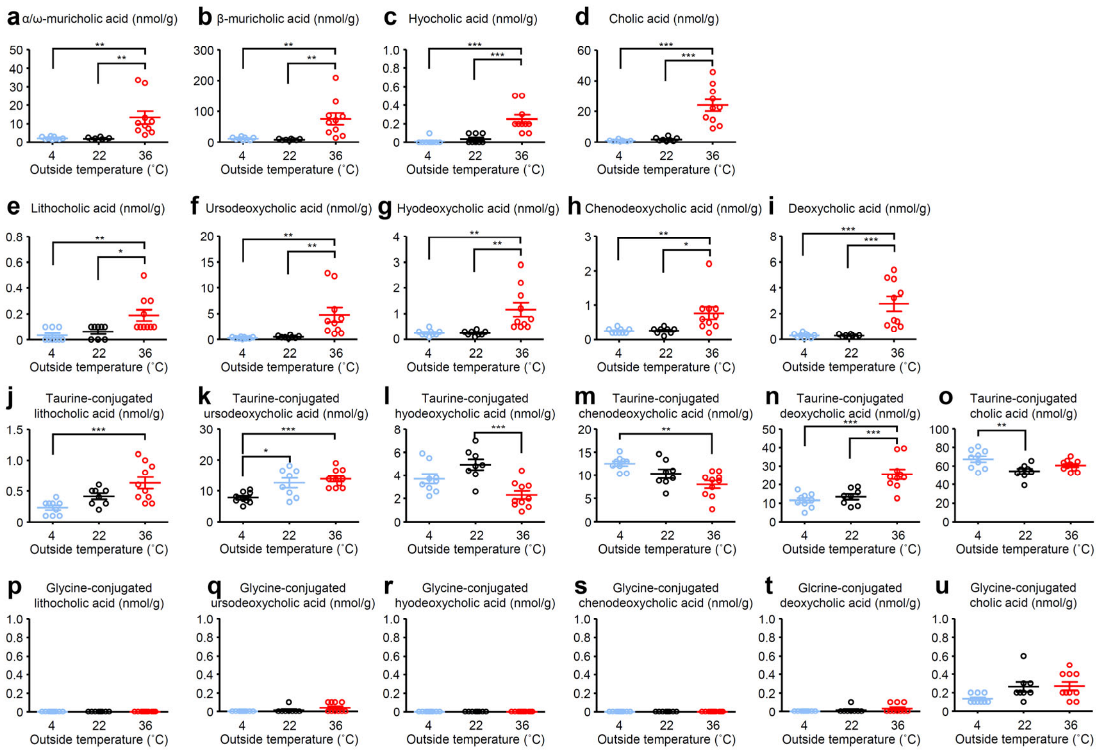

Supplementary Figure 16

The levels of bile acids in Liver.

a-u, Mice were kept at 4, 22, or 36 °C for 7 d. The levels of bile acids in liver were measured (4 °C, *n* = 9 mice; 22 °C, *n* = 8 mice; 36 °C, *n* = 10 mice). Data are mean ± s.e.m. Statistical significance was analysed by two-way analysis of variance (ANOVA) test. **P*<0.05, ***P*<0.01, ****P*<0.001.

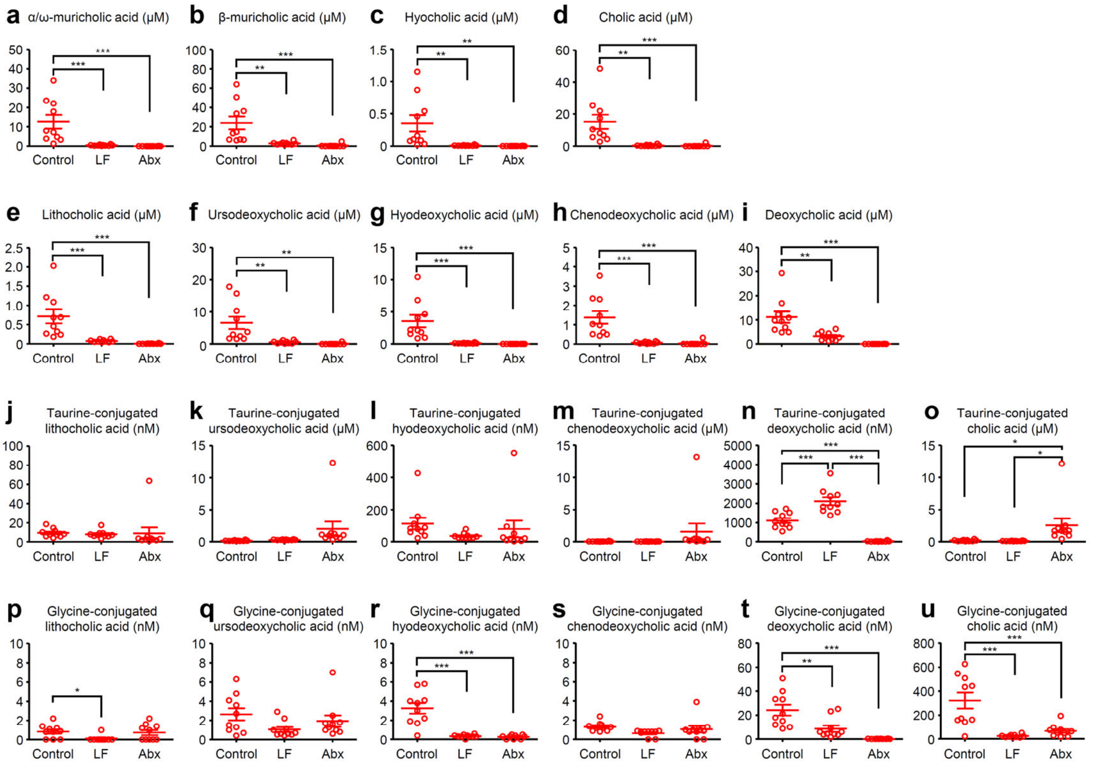

Supplementary Figure 17

The levels of bile acids in serum of high heat-exposed mice.

a-u, LF-fed, Abx-treated, and control mice were kept at 36 °C for 7 d. The levels of bile acids in serum were measured (control, *n* = 10 mice; LF, *n* = 10 mice; Abx, *n* = 10 mice). Data are mean ± s.e.m. Statistical significance was analysed by two-way analysis of variance (ANOVA) test. **P*<0.05, ***P*<0.01, ****P*<0.001.

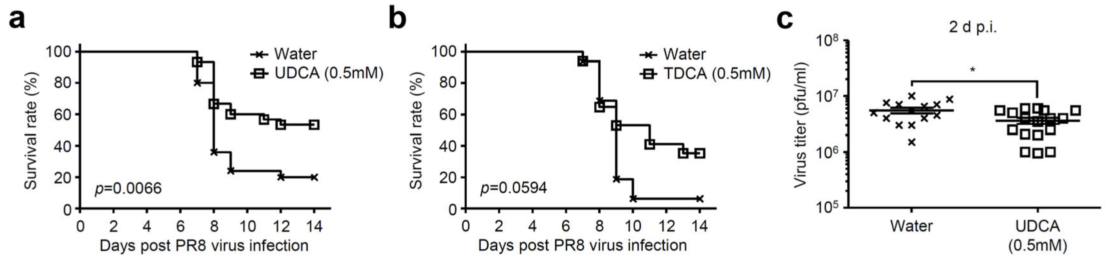

Supplementary Figure 18

Bile acids protect mice from influenza virus infection.

a-c, Room temperature-exposed mice given 0.5 mM of UDCA (**a, c**) or TDCA were infected intranasally with 1,000 pfu of influenza virus. Mortality (**a**, *n* = 25 mice for water-fed, *n* = 30 mice for UDCA-treated; **b**, *n* = 16 mice for water-fed, *n* = 17 mice for TDCA-treated) and virus titer in the lung wash (**c**; water, *n* = 14 mice; UDCA, *n* = 17 mice) were measured on indicated days after challenge. Data are mean ± s.e.m. Data are pooled from two independent experiments. Statistical significance was analysed by log-rank (Mantel-Cox) test (**a**, **b**), or two-tailed unpaired Student’s *t*-test (**c**; *p*=0.016282). *P<0.05.

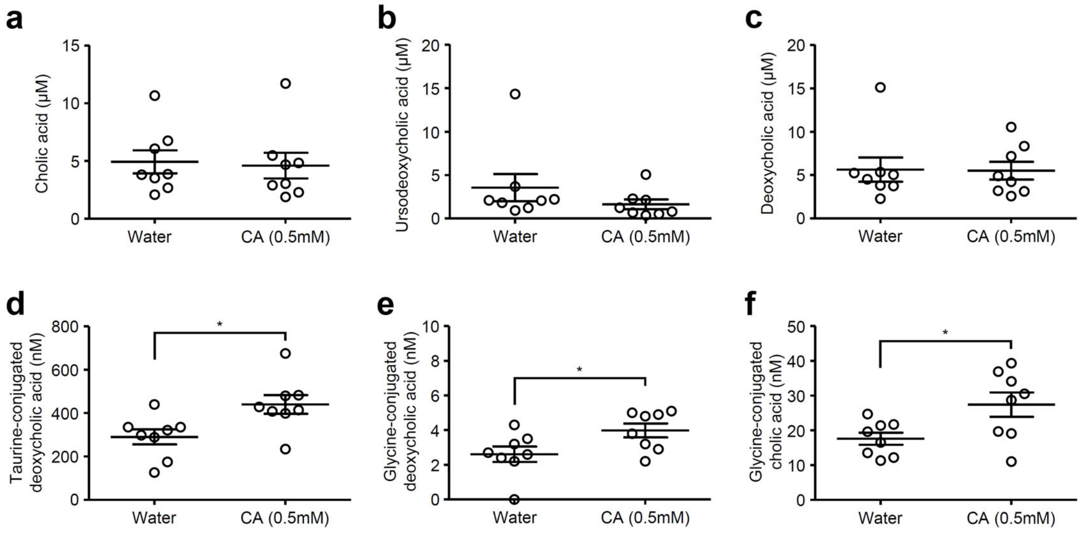

Supplementary Figure 19

The levels of bile acids in serum of control or CA-treated mice.

a-f, CA (0.5 mM)-treated or control mice were kept at 22°C for 7 d. The levels of bile acids in serum were measured (water, *n* = 8 mice; CA, *n* = 8 mice). Data are mean ± s.e.m. Statistical significance was analysed by two-tailed unpaired Student's *t*-test. **P*<0.05 (**d**; *p*=0.017031; **e**; *p*=0.036552; **f**; *p*=0.025267).

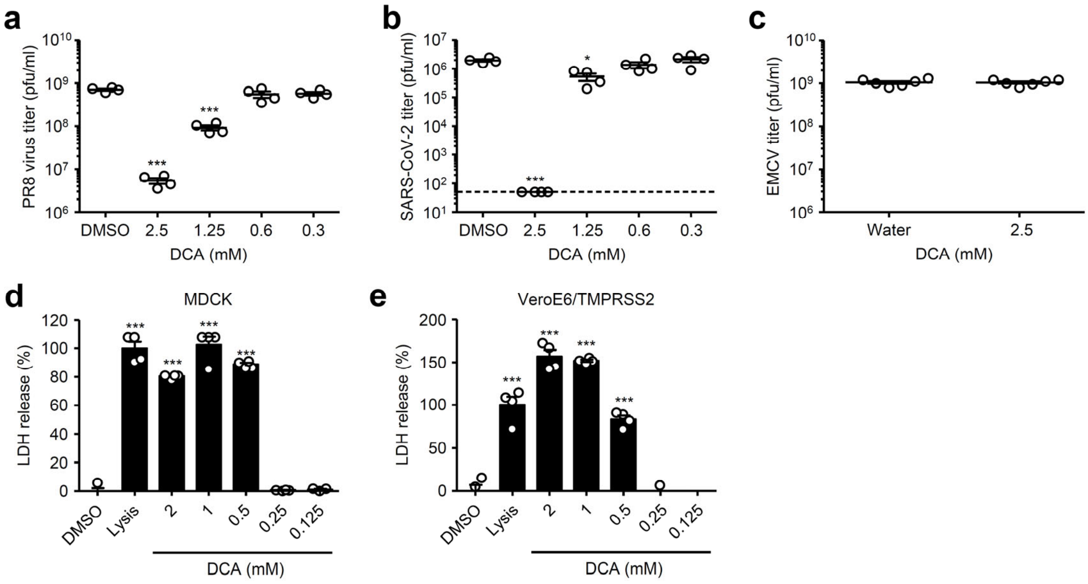

Supplementary Figure 20

Bile acids directly disrupt enveloped viruses.

a-c, Influenza virus (**a**), SARS-CoV-2 (**b**), and EMCV (**c**) were incubated with indicated amounts of DCA at 37 °C for 1 h. Virus titers were measured by standard plaque assay using MDCK (**a**; *n* = 4), VeroE6/TMPRSS2 (**b**; *n* = 4), or L929 cells (**c**; *n* = 6). **d, e,** Uninfected-MDCK (**d**; *n* = 4) or VeroE6/TMPRSS2 (**e**; *n* = 4) cells were cultured in the presence or absence of indicated amounts of DCA for 24 h. LDH activity was measured for cytotoxicity. Data are mean ± s.e.m. Data are representative of two independent experiments. Statistical significance was analysed by two-way analysis of variance (ANOVA) test. **P*<0.05, ****P*<0.001.

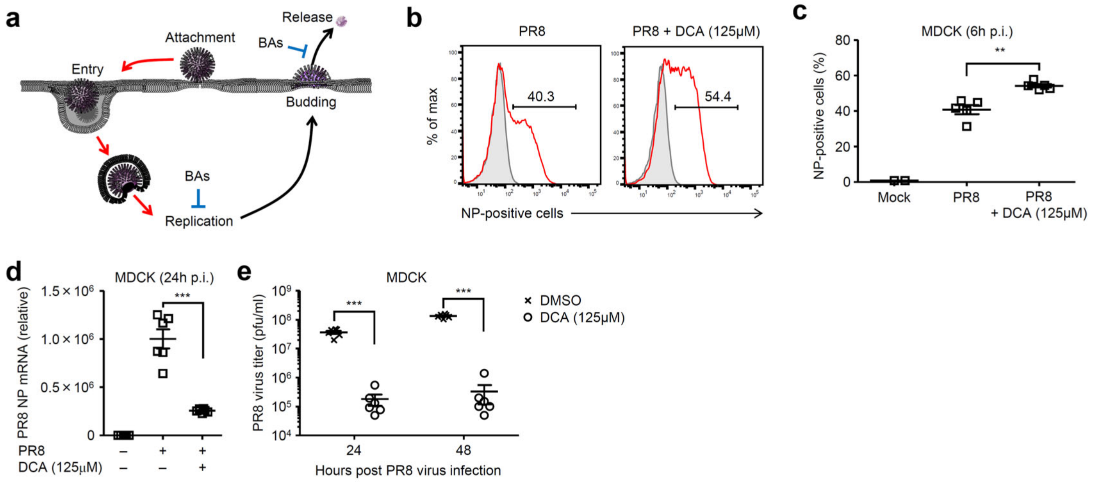

Supplementary Figure 21

Bile acids inhibit influenza virus replication.

a, Effects of bile acids (BAs) on influenza virus replication. Red arrows indicate enhancement. Blue blunt ended bars indicate inhibition. **b-e,** MDCK cells were infected with influenza virus in the presence or absence of 125 μM of DCA. Cells were collected at 6 h post infection, and intracellularly stained with nucleoprotein (NP)-specific antibody (**b**). Percentages of NP-positive cells are shown (**c**; mock, *n* = 2; PR8, *n* = 5; PR8 + DCA, *n* = 5). Total RNAs were extracted from uninfected or virus-infected cells at 24 h p.i. and influenza virus NP mRNA levels were assessed by quantitative reverse transcription PCR (**d**; *n* = 6). Cell-free supernatants were collected at 24 and 48 h p.i. and analyzed for virus titer by standard plaque assay using MDCK cells (**e**; *n* = 6). Data are mean ± s.e.m. Data are representative of two independent experiments. Statistical significance was analysed by two-way analysis of variance (ANOVA) test (**c**, **d**) or two-tailed unpaired Student's *t*-test (**e**). $^{**}P<0.01$, $^{***}P<0.001$ (**e**; 24 h p.i., $p=0.000007746$; 48 h p.i., $p=0.000000015$).

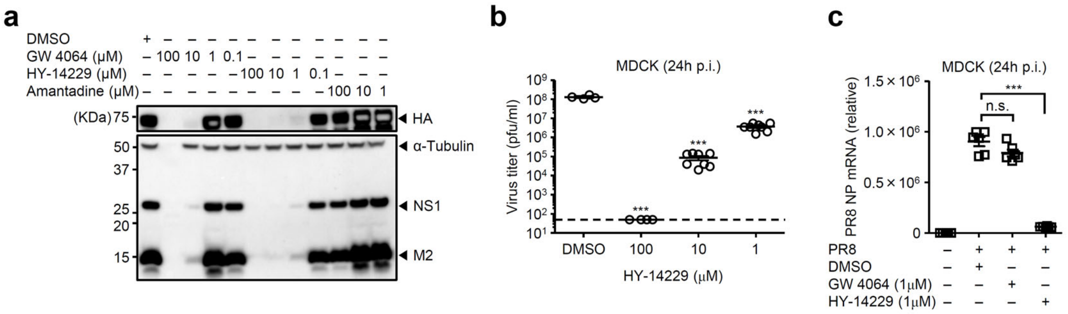

Supplementary Figure 22

Effects of TGR5 and FXR agonists on influenza virus replication.

a-c, MDCK cells were infected with influenza virus PR8 (an amantadine-resistant strain) in the presence or absence of indicated amounts of GW 4064, HY-14229, or amantadine. Cell lysates were collected at 24 h p.i. and analyzed by immunoblotting with indicated antibodies (**a**). Cell-free supernatants were collected at 24 h p.i. and analyzed for virus titer by standard plaque assay using MDCK cells (**b**; DMSO, n = 4; 100 μM, n = 4; 10 μM, n = 8; 1 μM, n = 8). Total RNAs were extracted from uninfected or virus-infected cells at 24 h p.i. and influenza virus NP mRNA levels were assessed by quantitative reverse transcription PCR (**c**; n = 6). Data are mean ± s.e.m. Data are representative of two independent experiments (**a**, **c**) or are pooled from two independent experiments (**b**). Statistical significance was analysed by two-way analysis of variance (ANOVA) test (**b**, **c**). ***P<0.001, n.s., not significant.

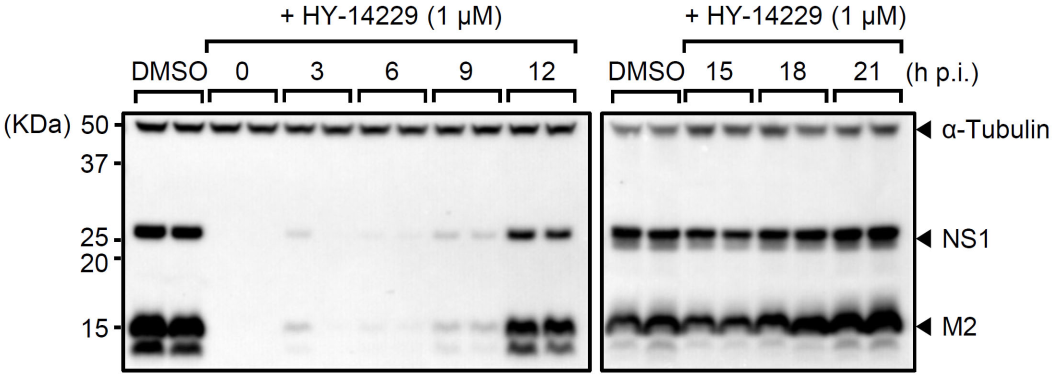

Supplementary Figure 23

Therapeutic effects of HY-14229 on influenza virus replication.

MDCK cells were infected with influenza virus PR8. After infection, the culture medium was replaced with medium with 1 µM of HY-14229 at indicated time points. Cell lysates were collected at 24 h p.i. and analyzed by immunoblotting with indicated antibodies. Data are representative of two independent experiments.

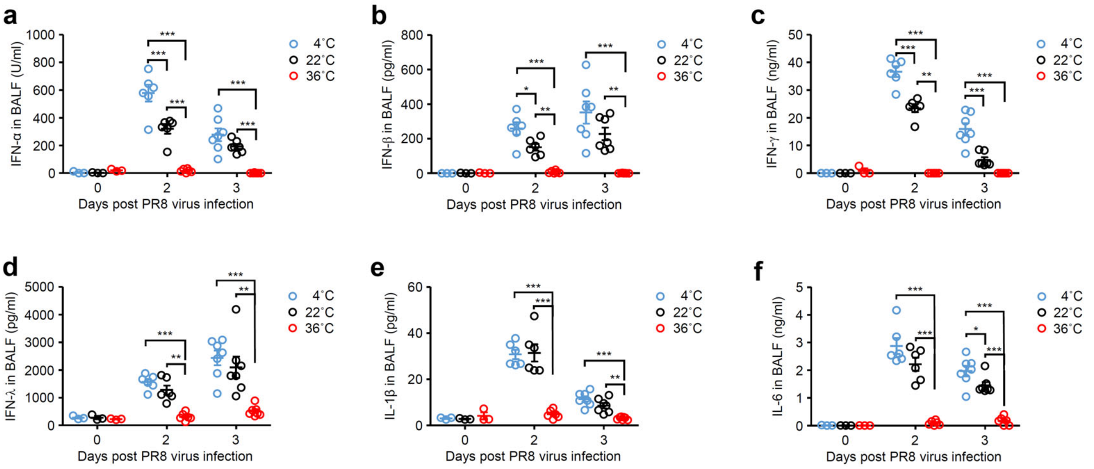

Supplementary Figure 24

Effects of outside temperature on influenza virus-induced cytokine production.

a-f, Mice were kept at 4, 22, or 36 °C for 7 d before influenza virus infection and throughout infection. Mice kept at 4, 22, or 36 °C were infected intranasally with 1,000 pfu of influenza virus. The lung washes were collected at indicated time points and analyzed for IFN-α (**a**), IFN-β (**b**), IFN-γ (**c**), IFN-λ (**d**), IL-1β (**e**), and IL-6 (**f**) (*n* = 3 mice at 0 d p.i., *n* = 6 mice at 2 d p.i., and *n* = 7 mice at 3 d p.i.). Data are mean ± s.e.m. Data are representative of two independent experiments. Statistical significance was analysed by two-way analysis of variance (ANOVA) test. *P<0.05, **P<0.01, ***P<0.001.

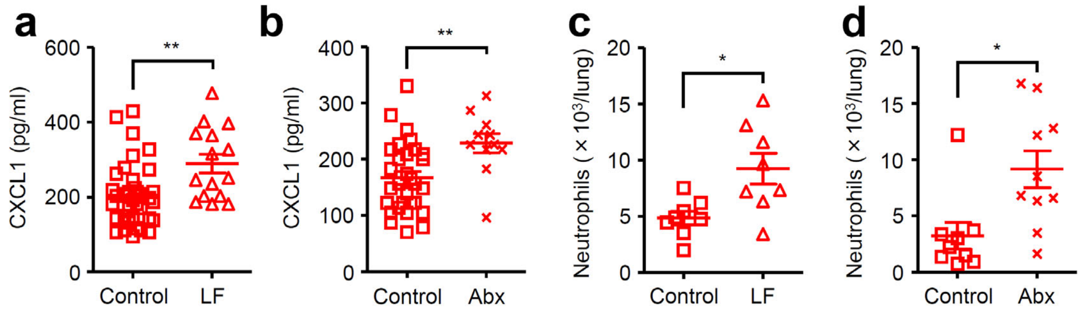

Supplementary Figure 25

Effects of outside temperature on influenza virus-induced cytokine production.

a-d, LF-fed, Abx-treated, and control mice kept at 36 °C were infected intranasally with 1,000 pfu of influenza virus. The lung washes were collected at 4 d p.i. and analyzed for CXCL1 by ELISA (**a**, *n* = 36 mice for control, *n* = 15 mice for LF-fed; **b**, *n* = 30 mice for control, *n* = 11 mice for Abx-treated). Leucocytes were isolated from the lung at 7 (**c**) or 9 (**d**) d p.i.. The number of Ly6C$^+$ Ly6G$^+$ neutrophils were analyzed by flow cytometry (**c**, *n* = 8 mice for control, *n* = 8 mice for LF-fed; **d**, *n* = 9 mice for control, *n* = 10 mice for Abx-treated). Data are mean ± s.e.m. Data are pooled from two independent experiments (**a**, **b**) or are representative of two independent experiments (**c**, **d**). Statistical significance was analysed by two-tailed unpaired Student's *t*-test. *P<0.05, **P<0.01 (**a**; p=0.003; **b**; p=0.0064; **c**; p=0.01131; **d**; p=0.01046;).

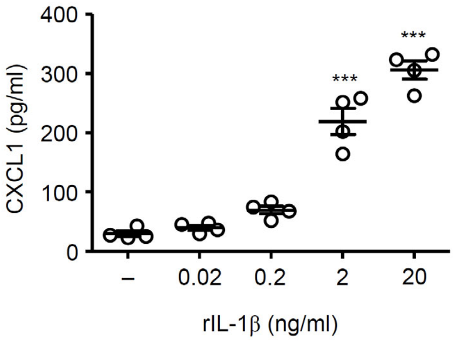

Supplementary Figure 26

Treatment of bone marrow-derived macrophages with IL-1β stimulates CXCL1 production.

bone marrow-derived macrophages were stimulated with indicated amounts of recombinant mouse IL-1β (rIL-1β). Cell-free supernatants were collected at 24 h post stimulation and analyzed for CXCL1 by ELISA (n = 4). Data are mean ± s.e.m. Data are representative of two independent experiments. Statistical significance was analysed by two-way analysis of variance (ANOVA) test. ***P<0.001.

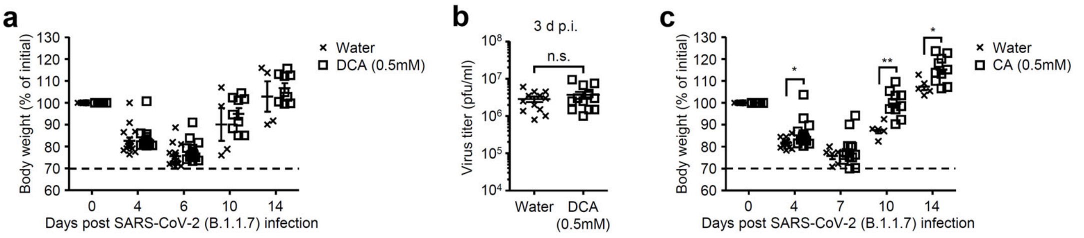

Supplementary Figure 27

Weight loss and virus titer of SARS-CoV-2-infected hamsters.

a-c, Room temperature-exposed hamsters given 0.5 mM of DCA (**a, b**) or CA were infected intranasally with 1.5×10^6 pfu of SARS-CoV-2 B.1.1.7 (alpha) variant. Weight loss (**a, c**; water, n = 16 hamsters; DCA, n = 14 hamsters; CA, n = 16 hamsters) and virus titer in the lung wash (**b**; water, n = 12 hamsters; DCA, n = 12 hamsters) were measured on indicated days after challenge. The dashed line indicates the limit of endpoint. Data are mean ± s.e.m. Data are pooled from two independent experiments. Statistical significance was analysed by two-tailed unpaired Student's *t*-test. *P<0.05, **P<0.01, n.s., not significant (**c**; 4 d p.i., p=0.013699; 10 d p.i., p=0.00111; 14 d p.i., p=0.022192).

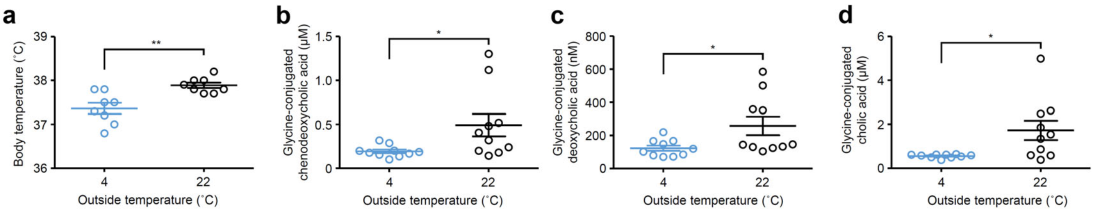

Supplementary Figure 28

Body temperature and the levels of bile acids in serum of cold- and room temperature-exposed hamsters.

a-d, Hamsters were kept at 4 or 22 °C for 7 d. Body temperature (**a**; 4 °C, *n* = 8 hamsters; 22 °C, *n* = 8 hamsters) and the levels of bile acids in serum (**b-d**; 4 °C, *n* = 10 hamsters; 22 °C, *n* = 10 hamsters) of naïve hamsters were measured. Data are mean ± s.e.m. Data are representative of two independent experiments (**a**). Statistical significance was analysed by two-tailed unpaired Student's *t*-test. *P<0.05, **P<0.01 (**a**; p=0.002226; **b**; p=0.032541; **c**; p=0.03274; **d**; p=0.016035).

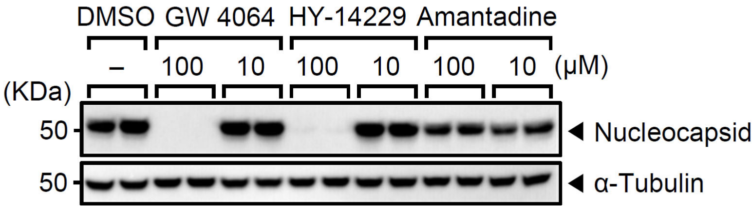

Supplementary Figure 29

Effects of TGR5 and FXR agonists on SARS-CoV-2 replication.

VeroE6/TMPRSS2 cells were infected with original SARS-CoV-2 (S-614D) in the presence or absence of indicated amounts of GW 4064, HY-14229, or amantadine. Cell lysates were collected at 24 h p.i. and analyzed by immunoblotting with indicated antibodies (*n* = 2). Data are representative of two independent experiments.

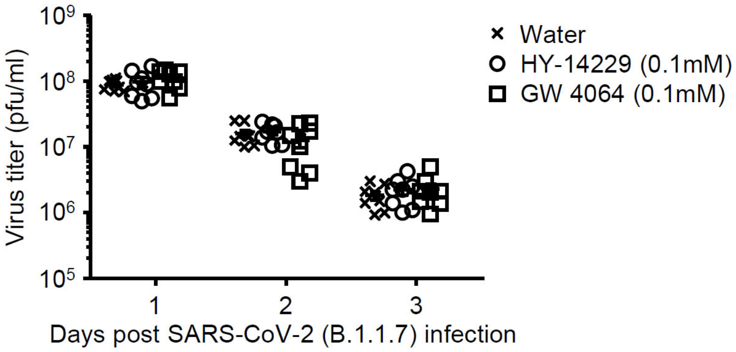

Supplementary Figure 30

Effects of TGR5 and FXR agonists on SARS-CoV-2 replication in hamsters.

Room temperature-exposed hamsters given 0.1 mM of HY-14229 or GW 4064 were infected intranasally with 1.5×10^6 pfu of SARS-CoV-2 B.1.1.7 (alpha) variant. Virus titers in the lung washes were measured on indicated days after challenge (water, *n* = 8 hamsters; HY-14229, *n* = 8 hamsters; GW 4064, *n* = 8 hamsters). Data are mean ± s.e.m. Data are pooled from two independent experiments. Statistical significance was analysed by two-way analysis of variance (ANOVA) test.

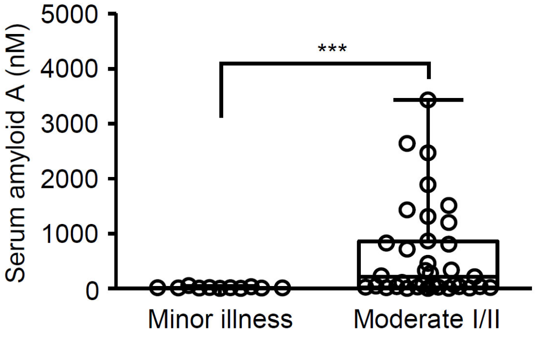

Supplementary Figure 31

Level of serum amyloid A in plasma of COVID-19 patients.

Concentrations of serum amyloid A in plasma of minor (n = 11 patients) versus moderate I/II (n = 35 patients) groups were measured. Each dot represents a unique patient. Data are mean ± s.e.m. Statistical significance was analysed by a two-tailed Mann-Whitney test. The centre line denotes the median value (50th percentile), while the white box contains the 25th to 75th percentiles of dataset. The black whiskers mark the 5th and 95th percentiles. ****P*<0.001 (*p*=0.0007).

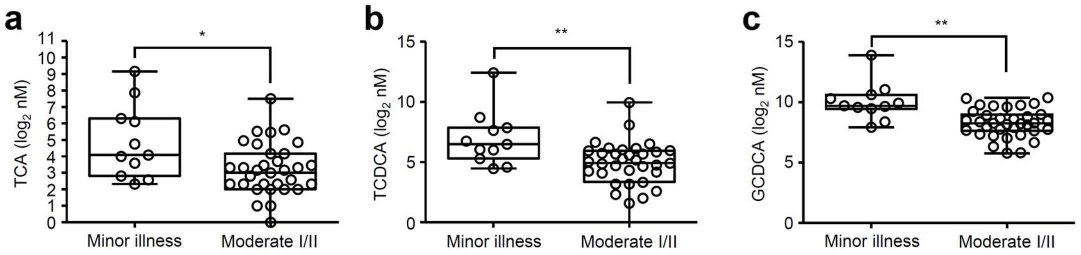

Supplementary Figure 32

Levels of bile acids in plasma of COVID-19 patients.

a-c, Concentrations of taurine-conjugated CA (TCA) (**a**), taurine-conjugated chenodeoxycholic acid (TCDCA) (**b**), and glycine-conjugated chenodeoxycholic acid (GCDCA) (**c**) in plasma of minor (n = 11 patients) versus moderate I/II (n = 35 patients) groups were measured. Each dot represents a unique patient. Data are mean ± s.e.m. Statistical significance was analysed by a two-tailed Mann-Whitney test. The centre line denotes the median value (50th percentile), while the white box contains the 25th to 75th percentiles of dataset. The black whiskers mark the 5th and 95th percentiles. *P<0.05, **P<0.01 (**a**; p=0.0143; **b**; p=0.0048; **c**; p=0.0012).

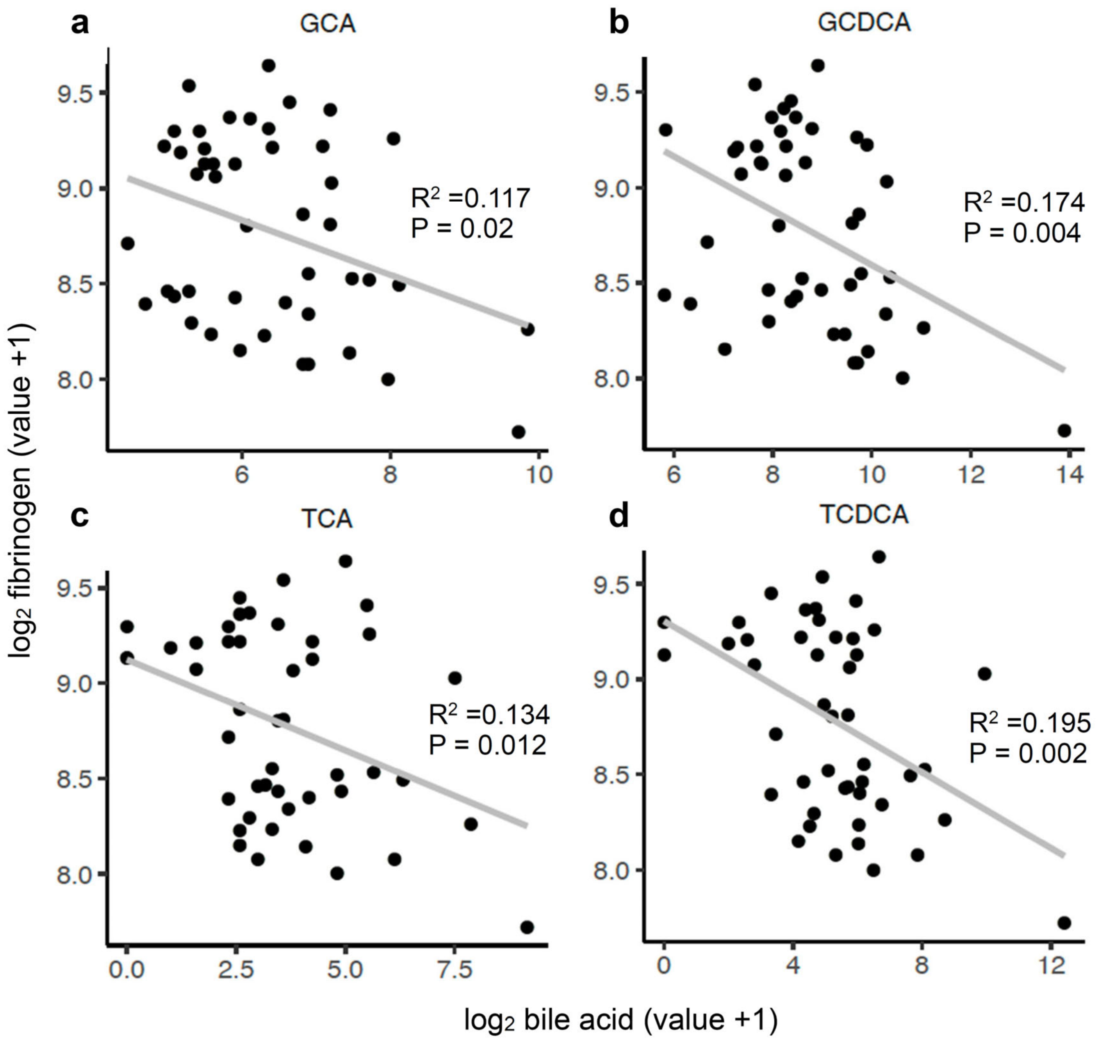

Supplementary Figure 33

The levels of plasma bile acids in COVID-19 patients are negatively correlated with the level of plasma fibrinogen as a biomarker of COVID-19 disease severity.

a-d, Scatterplots of the levels of plasma bile acids and a fibrinogen. Individual patients are represented as black circles. The solid gray line shows the regression line. R2 and P values were calculated based on the linear regression and two-sided Wald test, respectively. GCA: glycine-conjugated cholic acid, GCDCA: glycine-conjugated chenodeoxycholic acid, TCA: taurine-conjugated cholic acid, TCDCA: taurine-conjugated chenodeoxycholic acid.

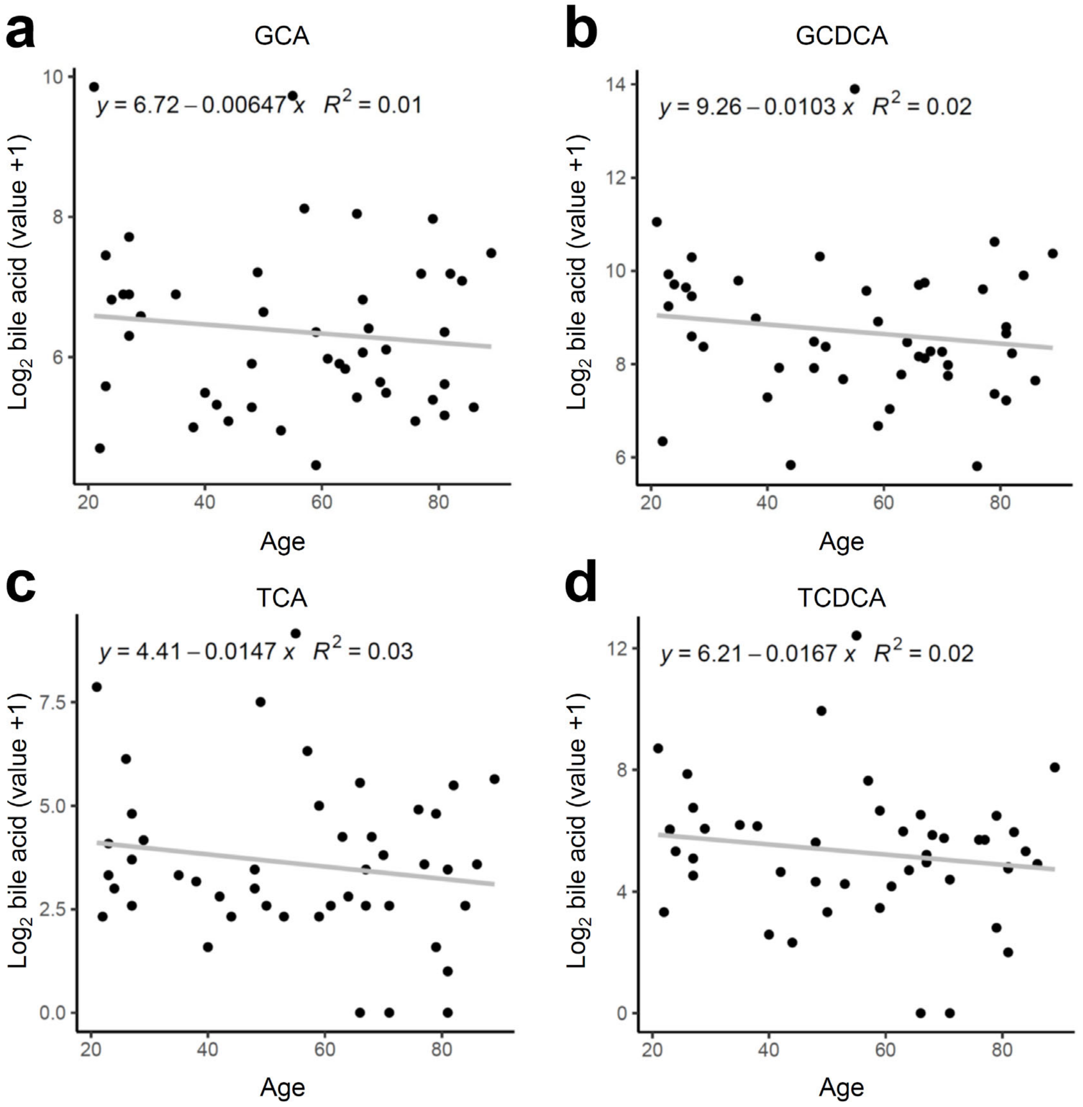

Supplementary Figure 34

The levels of plasma bile acids in COVID-19 patients are not correlated with the patients' age.

a-d, Scatterplots of the levels of plasma bile acids and patients' age. Individual patients are represented as black circles. The solid gray line shows the regression line. R2 was calculated based on the linear regression. GCA: glycine-conjugated cholic acid, GCDCA: glycine-conjugated chenodeoxycholic acid, TCA: taurine-conjugated cholic acid, TCDCA: taurine-conjugated chenodeoxycholic acid.

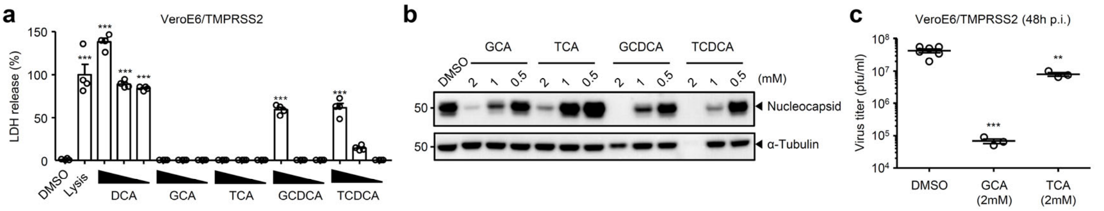

Supplementary Figure 35

GCA efficiently inhibits SARS-CoV-2 replication.

a, Uninfected-VeroE6/TMPRSS2 cells were cultured in the presence or absence of various amounts (2, 1, 0.5 mM) of DCA, GCA, TCA, GCDCA, or TCDCA for 24 h. LDH activity was measured for cytotoxicity (*n* = 4). **b, c**, VeroE6/TMPRSS2 cells were infected with original SARS-CoV-2 (S-614D) in the presence or absence of indicated amounts of GCA, TCA, GCDCA, or TCDCA. Cell lysates were collected at 24 h p.i. and analyzed by immunoblotting with indicated antibodies (**b**). Cell-free supernatants were collected at 48 h p.i. and analyzed for virus titer by standard plaque assay using VeroE6/TMPRSS2 cells (**c**; *n* = 6 tests for DMSO control, *n* = 3 tests for GCA and TCA). Data are mean ± s.e.m. Data are representative of two independent experiments. Statistical significance was analysed by two-way analysis of variance (ANOVA) test (**a**, **c**). ***P*<0.01, ****P*<0.001.

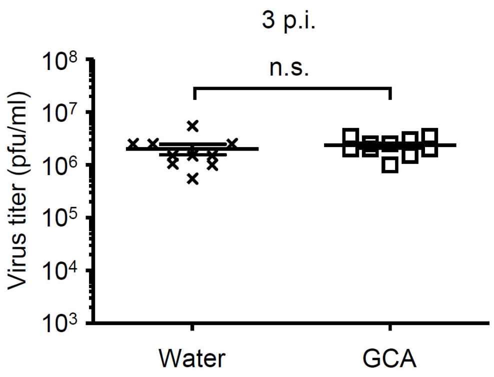

Supplementary Figure 36

Virus titer of SARS-CoV-2-infected hamsters.

Room temperature-exposed hamsters given 0.5 mM of GCA were infected intranasally with 1.5×10^6 pfu of SARS-CoV-2 B.1.1.7 (alpha) variant. Virus titer in the lung wash (water, *n* = 10 hamsters; DCA, *n* = 10 hamsters) were measured at 3 d p.i.. Data are mean ± s.e.m. Data are pooled from two independent experiments. Statistical significance was analysed by two-tailed unpaired Student’s *t*-test. n.s., not significant.

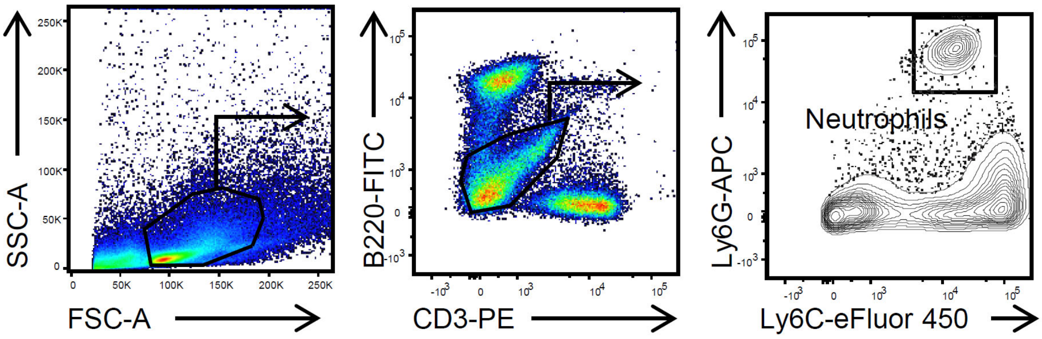

Supplementary Figure 37

Gating strategy for identifying neutrophils.

To identify neutrophil population, leucocytes were gated by forward and side scatter. Then, B cells and T cells were excluded based on B220 and CD3 expression, respectively. Ly6C and Ly6G double-positive cells were identified as neutrophils.

Supplementary Table 1. Demographic and background information for 46 patients.

Patient ID	Final symptom	Sex	Age	Ethnicity	Comorbidities	Hospitalization	Discharge	Duration
Tx01001	Minor illness	Female	27	Asian	Mycoplasma pneumonia	2020/11/7	2020/11/14	7
Tx01006	Minor illness	Male	24	Asian	Right little finger tendon rupture	2020/11/11	2020/11/21	10
Tx01008	Minor illness	Male	57	Asian	HIV	2020/11/12	2020/11/21	9
					Myocardial infarction			
					Hemangioma (base of the nose)			
					Hypertension			
					Dyslipidemia			
					Fatty liver			
Tx01010	Minor illness	Male	23	Asian	Herniated disc	2020/11/13	2020/11/26	13
Tx01018	Minor illness	Male	21	Asian	Asthma	2020/11/22	2020/12/1	9
Tx01020	Minor illness	Female	29	Asian	N/A	2020/11/20	2020/11/26	6
Tx01035	Minor illness	Male	55	Asian	Diabetes	2020/11/27	2020/12/24	27
					Liver cancer			
					Bacteremia			
					Colon polyps			
					Portal vein thrombosis			
					Peritonitis			
					Esophageal varices			
					Thyroid mass			
Tx01055	Minor illness	Female	79	Asian	Angina pectoris	2021/2/26	2021/3/9	11
					Dyslipidemia			

					Osteoporosis			
					Uterine fibroid			
					Fallopian tube obstruction disease			
Tx01058	Minor illness	Female	26	Asian	Pulmonary embolism	2021/3/11	2021/3/22	11
					Insomnia			
Tx01071	Minor illness	Male	42	Asian	Restless legs syndrome	2021/3/5	2021/3/15	10
					Hypertension			
					Sleep apnea syndrome			
Tx01079	Minor illness	Male	27	Asian	X-linked agammaglobulinemia	2021/2/14	2021/2/23	9
Tx01002	Moderate I	Male	53	Asian	Hepatitis B	2020/11/8	2020/11/20	12
Tx01011	Moderate I	Male	27	Asian	Familial Mediterranean fever	2020/11/14	2020/11/23	9
					Type 2 diabetes			
					Hypertension			
					Dyslipidemia			
					Obesity Type 3			
					Sleep apnea syndrome			
Tx01012	Moderate I	Male	49	Asian	Vagal reflex	2020/11/16	2020/11/22	6
					Dyslipidemia			
					Hypertension			
					Gout			
					Hyperuricemia			
Tx01015	Moderate I	Female	71	Asian	Type 2 diabetes	2020/11/18	2020/11/27	9
					Lumbar spondylolisthesis			

					Herpes zoster			
					Dyslipidemia			
Tx01021	Moderate I	Male	50	Asian	Gastric ulcer	2020/11/21	2020/11/28	7
Tx01023	Moderate I	Male	71	Asian	Diabetes mellitus	2020/11/23	2020/12/2	9
					Hypertension			
					Cholesteatoma			
Tx01025	Moderate I	Female	77	Asian	Appendicitis	2020/11/25	2020/12/11	16
					Kidney stones			
					Progressive supranuclear palsy			
Tx01027	Moderate I	Male	44	Asian	Asthma	2020/11/25	2020/12/8	13
Tx01032	Moderate I	Male	23	Asian	Influenza-associated encephalopathy	2020/11/27	2020/12/2	5
Tx01034	Moderate I	Female	66	Asian	Hypothyroidism	2020/11/29	2020/12/2	3
					Hypertension			
Tx01051	Moderate I	Female	79	Asian	Uterine fibroid	2020/12/24	2021/1/2	9
					Hypertension			
					Diabetes mellitus			
Tx01053	Moderate I	Male	22	Asian	X-linked agammaglobulinemia	2021/2/14	2021/2/23	9
					Pneumonia			
					Appendicitis			
Tx01054	Moderate I	Male	38	Asian	N/A	2021/2/26	2021/3/7	9
Tx01057	Moderate I	Female	68	Asian	Asthma	2021/2/2	2021/2/17	15
					Colon cancer			
					Cataract			

					Glaucoma			
Tx01059	Moderate I	Male	40	Asian	N/A	2020/12/1	2020/12/5	4
Tx01061	Moderate I	Male	35	Asian	N/A	2020/12/26	2021/1/8	13
Tx01064	Moderate I	Male	48	Asian	Alcoholic liver disease	2021/2/4	2021/2/24	20
					Asthma			
					Depression			
					Psoriasis vulgaris			
					Gout			
Tx01066	Moderate I	Female	63	Asian	Parkinson's disease	2020/12/11	2020/12/26	15
Tx01070	Moderate I	Male	61	Asian	Myocardial infarction	2020/12/26	2021/1/3	8
					Diabetes mellitus			
					Hypertension			
					Dyslipidemia			
Tx01074	Moderate I	Male	66	Asian	Diabetes mellitus	2020/12/25	2021/1/8	14
					Hypertension			
					Dyslipidemia			
					Hyperuricemia			
					Gout			
Tx01076	Moderate I	Female	81	Asian	Small cell lung cancer	2021/1/30	2021/2/16	17
					Diabetes mellitus			
					Hypertension			
					Arrhythmia			
					Neurosis			

Tx01077	Moderate I	Male	48	Asian	Hypertension	2021/2/27	2021/3/9	10
Tx01080	Moderate I	Female	89	Asian	Chronic renal failure<br>Asthma<br>Tuberculosis	2021/2/4	2021/2/15	11
Tx01082	Moderate I	Male	70	Asian	Herniated disc<br>Urinary retention<br>Prostatic hypertrophy	2020/12/4	2020/12/15	11
Tx01003	Moderate II	Male	59	Asian	Dyslipidemia<br>Sleep apnea syndrome<br>Hyperuricemia<br>Hemorrhoid<br>Fatty liver	2020/11/8	2020/11/21	13
Tx01013	Moderate II	Female	84	Asian	Hypertension	2020/11/16	2020/11/27	11
Tx01052	Moderate II	Male	82	Asian	Myocardial infarction<br>Dyslipidemia<br>Appendicitis<br>Hypertension<br>Right thigh abscess<br>Alcoholic liver disease<br>Diabetes mellitus	2021/1/18	2021/2/2	15
Tx01062	Moderate II	Male	81	Asian	Prostate cancer<br>Diverticular bleeding<br>Duodenal ulcer	2021/1/26	2021/2/20	25

					Cataract			
Tx01063	Moderate II	Female	76	Asian	T12/L1 Herniated disc<br>Hypertension<br>Asymptomatic cerebral infarction<br>Old T12 compression fracture	2020/12/6	2020/12/29	23
Tx01065	Moderate II	Male	64	Asian	N/A	2020/11/30	2020/12/8	8
Tx01067	Moderate II	Male	81	Asian	Dementia<br>Stroke sequelae<br>Hyperuricemia	2021/1/19	2021/1/31	12
Tx01069	Moderate II	Female	67	Asian	Dyslipidemia<br>Bilateral hip osteoarthritis	2021/1/17	2021/2/14	28
Tx01073	Moderate II	Female	86	Asian	Hypertension<br>Dyslipidemia	2020/12/6	2020/12/15	9
Tx01081	Moderate II	Male	59	Asian	Angina pectoris<br>Lumbar hernia<br>Mediastinal lymphadenopathy<br>Autonomic dysregulation<br>Gastric ulcer<br>High blood pressure	2021/2/17	2021/3/3	14
Tx01083	Moderate II	Male	67	Asian	Hypertension<br>Hyperuricemia<br>Prostate cancer	2021/5/14	2021/5/27	13

Uncropped scans of blots:

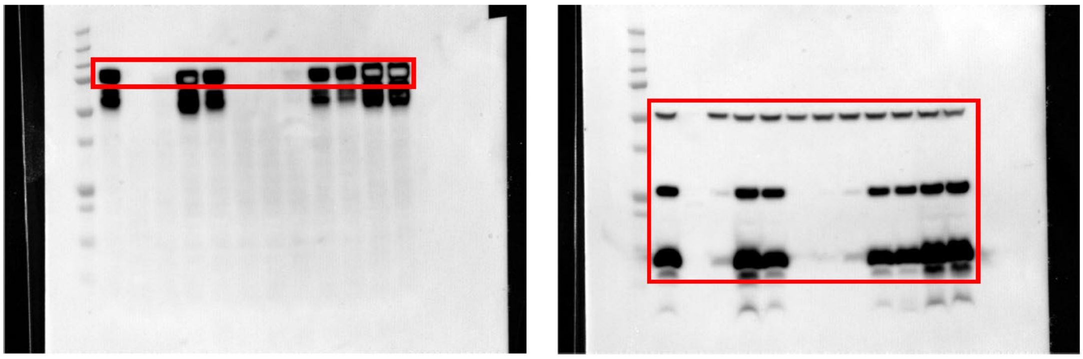

Supplementary Fig. 22a

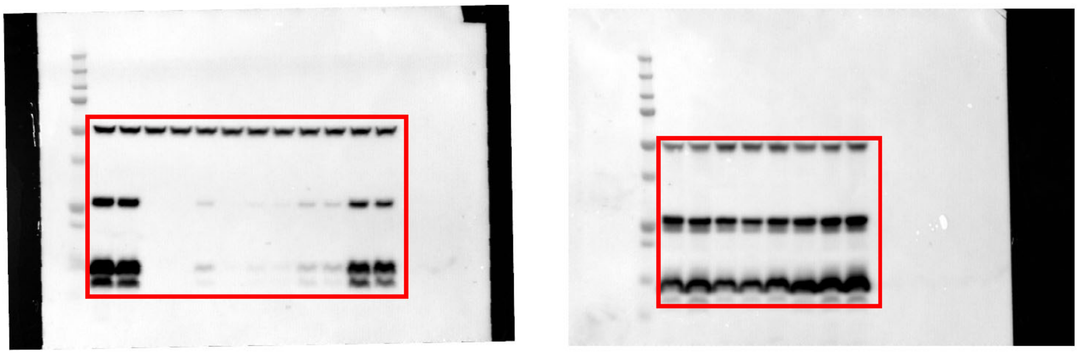

Supplementary Fig. 23

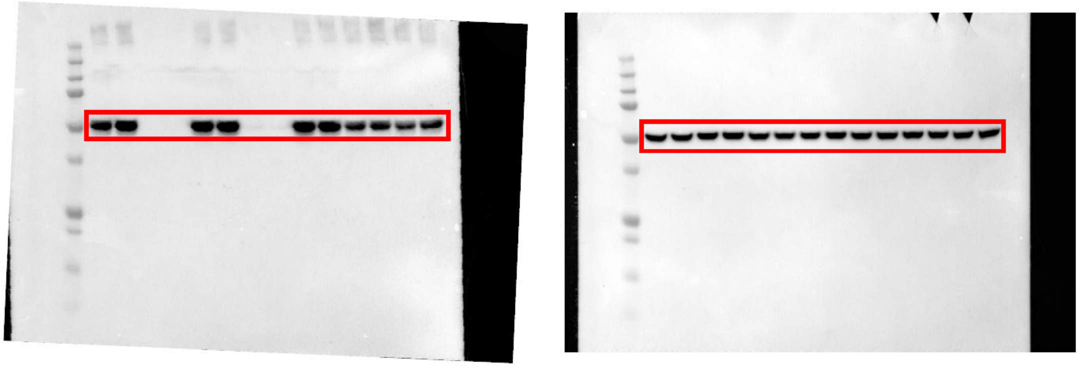

Supplementary Fig. 29

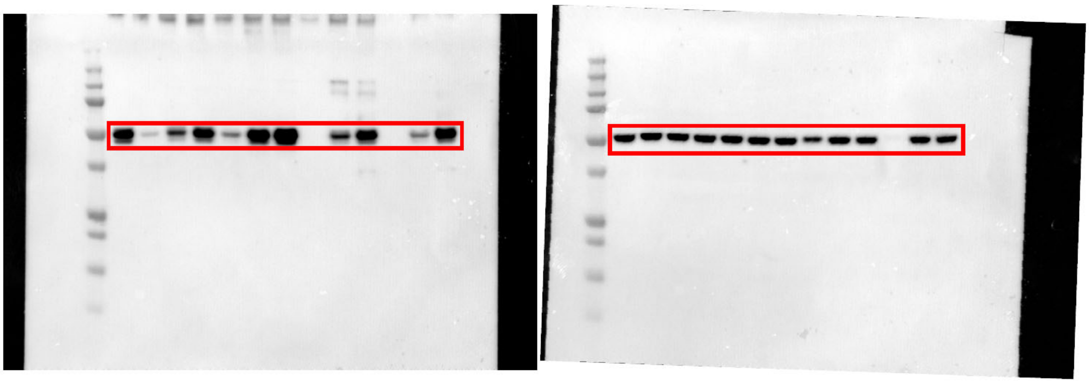

Supplementary Fig. 35b
