# Supplementary material for: High body temperature increases gut microbiota-dependent host resistance to influenza A virus and SARS-CoV-2 infection
Source: Nat Commun. 2023 Jun 30;14:3863. doi: 10.1038/s41467-023-39569-0 (PMC10313692; doi:10.1038/s41467-023-39569-0)
Supplement: Supplementary file 3 — Reporting Summary [file 41467_2023_39569_MOESM3_ESM.pdf]

nature portfolio

Corresponding author(s): Toshio Naito , Shinji Fukuda and Takeshi Ichinohe

Last updated by author(s): May 24, 2023

Reporting Summary

Nature Portfolio wishes to improve the reproducibility of the work that we publish. This form provides structure for consistency and transparency in reporting. For further information on Nature Portfolio policies, see our Editorial Policies and the Editorial Policy Checklist.

Statistics

For all statistical analyses, confirm that the following items are present in the figure legend, table legend, main text, or Methods section.

n/a	Confirmed	
☐	☒	The exact sample size (*n*) for each experimental group/condition, given as a discrete number and unit of measurement
☐	☒	A statement on whether measurements were taken from distinct samples or whether the same sample was measured repeatedly
☐	☒	The statistical test(s) used AND whether they are one- or two-sided<br>*Only common tests should be described solely by name; describe more complex techniques in the Methods section.*
☒	☐	A description of all covariates tested
☐	☒	A description of any assumptions or corrections, such as tests of normality and adjustment for multiple comparisons
☐	☒	A full description of the statistical parameters including central tendency (e.g. means) or other basic estimates (e.g. regression coefficient) AND variation (e.g. standard deviation) or associated estimates of uncertainty (e.g. confidence intervals)
☐	☒	For null hypothesis testing, the test statistic (e.g. *F*, *t*, *r*) with confidence intervals, effect sizes, degrees of freedom and *P* value noted<br>*Give P values as exact values whenever suitable.*
☒	☐	For Bayesian analysis, information on the choice of priors and Markov chain Monte Carlo settings
☒	☐	For hierarchical and complex designs, identification of the appropriate level for tests and full reporting of outcomes
☒	☐	Estimates of effect sizes (e.g. Cohen's *d*, Pearson's *r*), indicating how they were calculated

Our web collection on statistics for biologists contains articles on many of the points above.

Software and code

Policy information about availability of computer code

Data collection	FACSVerse flow cytometer (BD Biosciences)<br>Microplate Manager version 6 (Bio-Rad)<br>ImageQuant LAS 4000 Mini (GE Healthcare)<br>LightCycler 1.5 instrument (Roche Diagnostics)
Data analysis	FlowJo version 9.9.6 (Tree Star Inc.)<br>Microplate Manager version 6 (Bio-Rad)<br>ImageQuant TL (GE Healthcare)<br>LightCycler Data Analysis (GE Healthcare)<br>GraphPad Prism version 5

For manuscripts utilizing custom algorithms or software that are central to the research but not yet described in published literature, software must be made available to editors and reviewers. We strongly encourage code deposition in a community repository (e.g. GitHub). See the Nature Portfolio guidelines for submitting code & software for further information.

Data

Policy information about availability of data

All manuscripts must include a data availability statement. This statement should provide the following information, where applicable:

- Accession codes, unique identifiers, or web links for publicly available datasets
- A description of any restrictions on data availability
- For clinical datasets or third party data, please ensure that the statement adheres to our policy

The authors declare that the data supporting the findings of this study are available within the article and its Supplementary Information files, or are available on request. Source data are provided with this paper.

Human research participants

Policy information about studies involving human research participants and Sex and Gender in Research.

Reporting on sex and gender	Sex and gender were determined based on self-reporting.
Population characteristics	This is a case-control monocentric study that included 46 Japanese patients with COVID-19, hospitalized in the Juntendo University Hospital. Reverse transcription-polymerase chain reaction (RT-PCR) tests using nasal or pharyngeal swabs were performed for diagnosis of COVID-19. Electronic medical records of the patients' clinical characteristics on admission were collected. Data included self-reported symptoms before PCR testing, the patient's medical history, comorbidities, medical treatment received, blood examination result on admission, and chest x-ray and CT results. Data on COVID-19 management were collected as well. Self-reported symptoms on the day of the PCR test were collected via a questionnaire. All imaging and blood test results were collected on admission.
Recruitment	Hospitalized Japanese COVID-19 patients were recruited from October 2020 to March 2021 in Juntendo University Hospital.
Ethics oversight	This study was approved by the Ethics Committee of Juntendo University (No. H20-0222) and written informed consent was obtained from all participants.

Note that full information on the approval of the study protocol must also be provided in the manuscript.

Field-specific reporting

Please select the one below that is the best fit for your research. If you are not sure, read the appropriate sections before making your selection.

☒ Life sciences ☐ Behavioural & social sciences ☐ Ecological, evolutionary & environmental sciences

For a reference copy of the document with all sections, see nature.com/documents/nr-reporting-summary-flat.pdf

Life sciences study design

All studies must disclose on these points even when the disclosure is negative.

Sample size	All experimental sample sizes were determined according to the previous published reports.
Data exclusions	No data were excluded from the analysis
Replication	All experiments depicted included at least three biological replicates and were representative of at least 2 experimental replicates
Randomization	In Figure 1e and f, allocation into the experimental group was not random because the experiments were performed in 8-, 52-, 101-weeks-old female mice.. All other WT mice and hamsters were randomly assigned to experimental groups.
Blinding	All animal studies were not blinded since treatment and experimental analysis could not be separated, blinding of the investigators was not possible.

Reporting for specific materials, systems and methods

We require information from authors about some types of materials, experimental systems and methods used in many studies. Here, indicate whether each material, system or method listed is relevant to your study. If you are not sure if a list item applies to your research, read the appropriate section before selecting a response.

Materials & experimental systems

n/a	Involved in the study	
☐	☒	Antibodies
☐	☒	Eukaryotic cell lines
☒	☐	Palaeontology and archaeology
☐	☒	Animals and other organisms
☐	☒	Clinical data
☒	☐	Dual use research of concern

Methods

n/a	Involved in the study	
☒	☐	ChIP-seq
☐	☒	Flow cytometry
☒	☐	MRI-based neuroimaging

Antibodies

Antibodies used: Monoclonal antibodies against influenza A virus M2 protein (14C2, Cat#sc-32238; 1:1,000) and α tubulin (DM1A, sc-32293; 1:2,000) were purchased from Santa Cruz Biotechnology. Rabbit polyclonal antibody against SARS-CoV-2 nucleocapsid protein (Cat#33336; 1:1,000) were purchased from Cell Signaling Technology (Danvers, MA, USA). Horseradish peroxidase-conjugated anti-murine IgG antibody (Cat#115-035-003) was purchased from Jackson Immuno Research Laboratories. Horseradish peroxidase-conjugatedanti-rabbit IgG antibody (Cat#G-21234) was purchased from Invitrogen.

Validation: All antibodies used in this study are commercially available, and were used with validation procedures by the manufacturers.

Eukaryotic cell lines

Policy information about cell lines and Sex and Gender in Research

Cell line source(s): Madin-Darby canine kidney (MDCK) cells and VeroE6 cells stably expressing transmembrane protease serine 2 (VeroE6/TMPRSS2) were purchased from JCRB Cell Bank (Osaka, Japan).

Authentication: Cell lines were authenticated by morphology

Mycoplasma contamination: Cell lines were routinely tested for mycoplasma contamination, and negative for mycoplasma contamination.

Commonly misidentified lines (See ICLAC register): No commonly misidentified cell lines were used.

Animals and other research organisms

Policy information about studies involving animals; ARRIVE guidelines recommended for reporting animal research, and Sex and Gender in Research

Laboratory animals: Six-week-old female C57BL/6J mice and 4-week-old female Syrian hamsters obtained from Japan SLC, Inc. were used as WT controls. For some experiments we used aged (52- to 122-week-old) female C57BL/6J mice obtained from CLEA Japan, Inc. (Fig. 1e, f and Supplementary Fig. 7 and 10). B6.Cg-Tg(K18-ACE2)2Prlmn/J (K18-hACE2) mice were purchased from The Jackson Laboratory and subsequently bred at The University of Tokyo. Six-week-old female B6.Cg-Tg(K18-ACE2)2Prlmn/J (K18-hACE2) mice were used in this study.

Wild animals: No wild animals were used in the study.

Reporting on sex: Six-week-old female C57BL/6J mice and 4-week-old female Syrian hamsters were used in this study.

Field-collected samples: No field collected samples were used in the study.

Ethics oversight: All animal experiments were performed in accordance with University of Tokyo's Regulations for Animal Care and Use, which were approved by the Animal Experiment Committee of the Institute of Medical Science, the University of Tokyo.

Note that full information on the approval of the study protocol must also be provided in the manuscript.

Clinical data

Policy information about clinical studies

All manuscripts should comply with the ICMJEguidelines for publication of clinical research and a completedCONSORT checklist must be included with all submissions.

Clinical trial registration: This study was approved by the Ethics Committee of Juntendo University (No. H20-0222).

Study protocol	This is a case-control monocentric study that included 46 patients with COVID-19, hospitalized in the Juntendo University Hospital. Reverse transcription-polymerase chain reaction (RT-PCR) tests using nasal or pharyngeal swabs were performed for diagnosis of COVID-19. Electronic medical records of the patients' clinical characteristics on admission were collected. Data included self-reported symptoms before PCR testing, the patient's medical history, comorbidities, medical treatment received, blood examination result on admission, and chest x-ray and CT results. Data on COVID-19 management were collected as well. Self-reported symptoms on the day of the PCR test were collected via a questionnaire. All imaging and blood test results were collected on admission.
Data collection	All hospitalized patients in Juntendo University Hospital were recruited from October 2020 to March 2021 and clinical and plasma bile acids data were collected until December 2021.
Outcomes	The classification of severity of COVID-19 patients was based on Ministry of Health, Labour and Welfare criteria (oxygen saturation, clinical symptoms, and imaging findings).

Flow Cytometry

Plots

Confirm that:

- [x] The axis labels state the marker and fluorochrome used (e.g. CD4-FITC).
- [x] The axis scales are clearly visible. Include numbers along axes only for bottom left plot of group (a 'group' is an analysis of identical markers).
- [x] All plots are contour plots with outliers or pseudocolor plots.
- [x] A numerical value for number of cells or percentage (with statistics) is provided.

Methodology

Sample preparation	For neutrophil staining, single-cell suspensions of lung samples were incubated with APC-labeled anti-Ly6G (Invitrogen, #17-9668-82; 1:100) and eFluor 450-labeled anti-Ly6C (Invitrogen, #48-5932-82; 1:100). For the detection of influenza virus-infected cells, MDCK cells were fixed and permeabilized using a Cytofix/Cytoperm kit (BD Biosciences, 554714), and intracellulary stained with FITC-labeled mouse anti-influenza virus NP (abcam, #ab20921; 1:100) antibody.
Instrument	FACSVerse flow cytometer (BD Biosciences)
Software	Flowjo v9.9.6 (Tree star Inc.)
Cell population abundance	No cell sorting procedure was used in this study.
Gating strategy	Cell debris were excluded by SSC-A and FSC-A gating. Doublets were excluded by FSC-H and FSC-W gating and SSC-H and SSC-W for all flow cytometry analysis.

- [x] Tick this box to confirm that a figure exemplifying the gating strategy is provided in the Supplementary Information.
